# Interplay between geometry, electron density, and polarizability of the controversial drug atoxyl in crystal and biological environments

**DOI:** 10.1039/d5ra05936d

**Published:** 2025-11-27

**Authors:** Yaser Balmohammadi, Eduardo Metry, Lorraine A. Malaspina, Georgia Cametti, Yuiga Nakamura, Simon Grabowsky

**Affiliations:** a University of Bern, Department of Chemistry, Biochemistry and Pharmaceutical Sciences Freiestrasse 3 3012 Bern Switzerland simon.grabowsky@unibe.ch; b University of Bern, Institute of Geological Sciences Baltzerstrasse 3 3012 Bern Switzerland; c Japan Synchrotron Radiation Research Institute (JASRI) Sayo-cho Hyogo 679-5198 Japan

## Abstract

The molecule *para*-arsanilic acid was historically used and misused as a drug under the name atoxyl. It is a biologically active compound in the class of arsenic-organic molecules. In this study, we aim for an understanding of its mode of action with particular focus on the role of the arsenic atom in the structure. We carried out experimental quantum crystallographic studies of *p*-arsanilic acid in its zwitterionic form and found that there is a rich network of intermolecular interactions in the small-molecule crystal structure that resembles interactions in the biological environment. However, the arsenic atom is not involved in these interactions, but the internal polarization of the molecule by its environment is governed by the large polarizability of the As–C bond. By adopting different strategies to simulate the interaction density and the interaction electrostatic potential, we delineate effects of conformation and geometry from the pure polarization of the molecule by its neighbors.

## Abstract 2

Arsenic is infamous for its toxicity; and yet, As compounds such as atoxyl have been used and misused for therapeutic purposes. Here, we use quantum crystallography to understand atoxyl's behavior in different environments.

## Graphical abstract

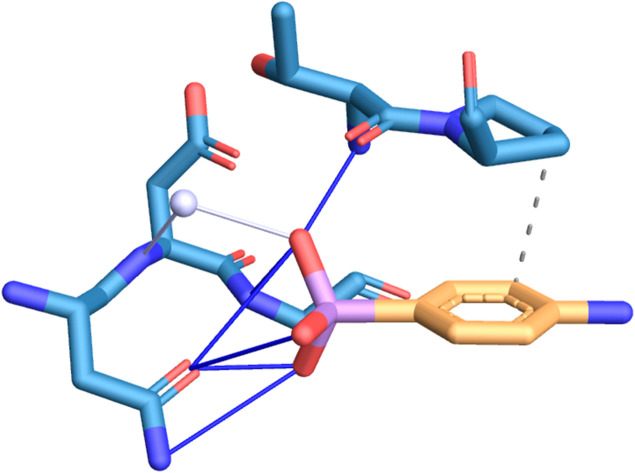

## Introduction

The element arsenic is known for its toxicity in its elemental form and in both oxidation states As(iii) (as arsenite) and As(v) (as arsenate).^1^ Historically, arsenic was used in warfare and assassination.^2^ Persian metallurgists (6–4th century BC) reportedly used it to poison enemy water supplies.^2^ In the 15th and 16th centuries, the Borgia family employed an arsenic compound called “La Cantarella” to eliminate rivals.^3^ Additionally, high arsenic levels found in Napoleon Bonaparte's hair suggest that he suffered from chronic arsenic poisoning.^4^ Despite its toxic reputation, arsenic has long been used therapeutically. Dating back over 2400 years, Greek and Chinese practitioners utilized arsenic compounds like realgar and orpiment for treating ulcers and malaria-related fevers.^5^ Arsenic trioxide, the active ingredient in the traditional Chinese medicine pishuang, has been used for centuries and was shown in the 1970s to effectively treat acute promyelocytic leukemia (APL).^6^ Historically, arsenic-based treatments have targeted conditions like psoriasis, syphilis, rheumatism, trypanosomiasis, and leukemia.^7–12^ More recently, darinaparsin showed potential against SARS-CoV-2 in an *in silico* study.^13^ In this study, we focus on the most controversial historic arsenic-based drug atoxyl to explore its structure and electron density using quantum crystallography.

In 1863, French scientist Antoine Béchamp synthesized a new arsenic compound by mixing aniline with arsenic acid, naming it “un anilide de l'acide arsénique”.^14^ Interest in the compound, later known as atoxyl, resurfaced around 1902 when Walther Schild suggested its medical use.^15^ In the following years, researchers explored atoxyl's potential in treating skin conditions like lichen ruber, psoriasis, and pemphigus vulgaris,^15–17^ with mixed results.^18^ Sleeping sickness in Africa, caused by a parasite transmitted by the Tsetse fly, became a concern for imperial Germany, particularly in its colony “German East Africa” (now Burundi, Rwanda, mainland Tanzania, and part of Mozambique). To combat the disease, doctors—including Robert Koch and Friedrich–Karl Kleine—were dispatched, focusing their efforts around Lake Victoria and the Ssese Islands.^19–21^ Atoxyl was tested as a treatment, and while some patients initially improved, the parasite persisted. Koch increased the dosage, leading to severe side effects such as pain, shivering, and even blindness. Eventually, atoxyl was replaced by more effective treatments. Despite being too toxic for human use, atoxyl was repurposed in livestock feed to promote growth and combat parasites. Most of the arsenic is excreted, but some remains in the meat, and the rest can leach into groundwater, eventually reaching drinking water. Due to these risks, many countries have banned its agricultural use. In 2013, the U.S. FDA also banned atoxyl in poultry and pork production.^22^

**Scheme 1:**
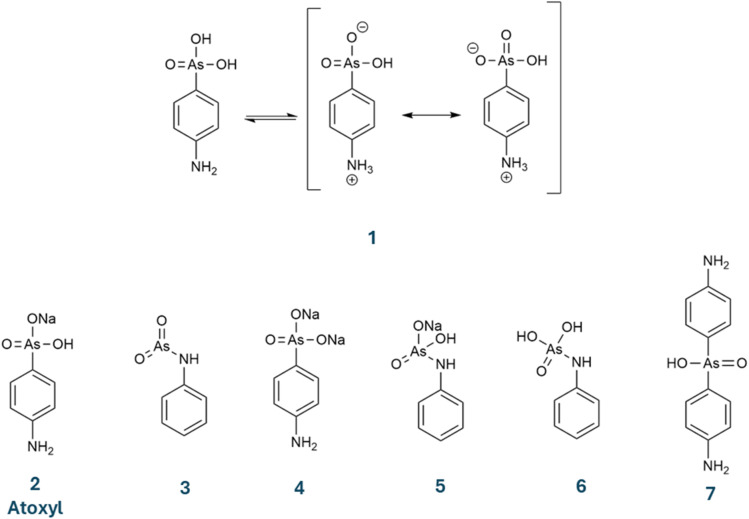
Chemical formulas suggested for atoxyl over the years (1–7). In this study, we investigate *p*-arsanilic acid in its zwitterionic form (1).

The molecular formula that was attributed to atoxyl has evolved over time. Béchamp originally proposed C_12_H_8_AsNO_6_, although he did not assign a structure. As the inventor of a cost-effective method for synthesizing aniline, Béchamp may have envisioned a doubly substituted arsenic acid—similar to a compound later synthesized by Kober in 1909 (molecule 7 in Scheme 1).^23^ Throughout the early 20th century, several alternative structures were proposed, as shown in Scheme 1 (molecules 3–6).^24^ Ultimately, the sodium salt 2 became the accepted structure for atoxyl. Ehrlich identified a free amino group due to its ability to undergo diazotization.^25^ The arsenic group was placed in the *para* position based on substitution reactions with iodine. The ambiguity of defining atoxyl remains, as shown in two papers by Riethmiller: in 1999 (ref. 26) he referred to it as the sodium salt (2), and in 2005 (ref. 27) as the sodium-free neutral molecule (1). The compound analyzed in this paper is the sodium-free, zwitterionic *p*-arsanilic acid (1). We refrain from using the unclear term atoxyl further in this paper but use the term *p*-arsanilic acid.

The mode of action of an active pharmaceutical ingredient (API) typically involves intermolecular interactions with proteins/enzymes in a biological environment. Upon docking to the protein, the API undergoes changes in conformation and polarization of its electron-density distribution due to specific interactions with protein residues.^28–32^ To model these changes during the biological recognition process experimentally, the crystalline environment in the small-molecule crystal structure has been shown to simulate the biological context to some extent, as the intermolecular interactions in both environments are similar, allowing for an approximation of the API's behavior *in vivo*.^30–35^ Intermolecular interactions involving *p*-arsanilic acid have been shown to be important not only in the biological context, but also in matrices to filter arsenic from freshwater.^36^

Computationally, the electron-density distribution of a small molecule can be estimated across various clearly defined environments, such as the isolated state, solutions, crystalline and biological states.^37^ The difference between the electron density of an isolated molecule in the vacuum and that of the same molecule in a crystalline environment is termed “interaction density”.^38^*In silico*, it is the difference between the quantum-mechanical electron densities from isolated-molecule and periodic-boundary condition calculations.^39–41^ Experimentally, it can be modelled from X-ray diffraction structure factors, usually employing the multipole model.^42–46^ Another approach involves comparing non-fitted and fitted wavefunctions through the X-ray constrained wavefunction fitting (XCW) procedure.^47,48^ In this framework, the non-fitted wavefunction represents the electron density of the molecule in isolation, while the fitted wavefunction corresponds to its polarized state in a crystalline environment.

The interaction density has been proposed as a measure for drug–receptor interactions, offering insights into the redistribution of a drug molecule's electron density within the receptor's active site.^49^ Besides the electron-density distribution, in drug design, evaluating the electrostatic potential is crucial for assessing the compatibility of a drug molecule with the protein binding pocket. It provides more direct insight into the biological recognition process of the drug. Since electron density and electrostatic potential are intrinsic properties derived from the wavefunction, we also analyze the “interaction electrostatic potential” (interaction ESP)—defined as the difference in electrostatic potential between the isolated molecule and the molecule in a crystalline environment.^32^

## Experimental and computational section

### Definitions and background

Polarity describes the uneven distribution of electron density within a bond or molecule, resulting in partial positive (δ^+^) and partial negative (δ^−^) charges. This charge separation gives rise to a molecular dipole moment that can be calculated from the bond contributions using the formula^50–54^
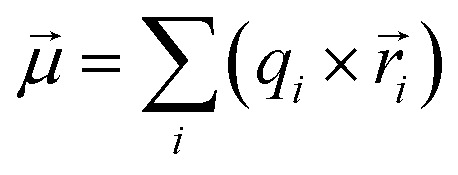
, in which *q*_*i*_ is the absolute amount of the charge at either end of the bond *i*, *r⃑*_*i*_ is the distance vector for that charge separation in bond *i*, and *μ⃗* is the dipole moment vector of the molecule. A molecule can be nonpolar despite having polar bonds if the bond dipole moments cancel out due to molecular symmetry. This illustrates that both the polarization of the electron-density distribution and the bond geometry (*i.e.*, conformation) influence the overall dipole moment of a drug molecule in a specific environment.

The electric polarizability is the molecule's ability to adjust to external electric fields. This flexibility enables a molecule to adapt its electronic structure to different environments. Electric polarizability refers to the shift of the electron cloud relative to the nuclei, inducing a dipole moment. The first-order (linear) electric polarizability describes the molecule's linear response to such an external field:^55–57^*p⃑* = α × *E⃑*. *p⃑* is the induced electric dipole moment, *α* is the electric polarizability (a scalar or tensor, depending on the system), and *E⃑* is the external electric field. The induced dipole moment increases proportionally with the applied electric field, making first-order polarizability dominant under weak fields. It plays a key role in linking electron density to optical properties like refraction and Raman scattering. In strong fields or complex systems, higher-order (nonlinear) effects may appear,^56^ but here we focus on first-order polarizability.

To calculate atomic polarizabilities, we must first define atomic volumes using a partitioning scheme. We used the PolaBer software,^55^ which applies Bader partitioning from quantum theory of atoms in molecules (QTAIM).^58^ PolaBer computes polarizability tensors by applying small electric fields and analyzing the resulting changes in the partitioned electron density. This requires calculating *p*-arsanilic acid's wavefunction both without an electric field and under small fields (±*X*, ±*Y*, ±*Z*), ensuring the field strength stays below 0.005 a.u. to maintain a linear response.

### Strategies

We propose two strategies to study how various structural and electronic settings influence *p*-arsanilic acid, particularly focusing on the interplay between geometry/conformation, polarizability, and the polarization of the electron-density distribution.

#### Strategy 1: fixed geometry

In this approach, the geometry of *p*-arsanilic acid is fixed, obtained from the X-ray diffraction experiment *via* the quantum-crystallographic refinement technique Hirshfeld atom refinement (HAR).^59,60^ Using this fixed geometry, we alter the model of the electron density and the environment, so that the polarization of the electron density is changed. Since the geometry remains constant, the dipole moment vector's distance parameter is also fixed, so only charge differences are of relevance in this strategy. XCW refers to X-ray constrained wavefunction fitting^61–63^ in the variant that is based on Hirshfeld atoms.^64^ Models in Strategy 1 are summarized below; more details are given in the section "Experimental details":

1. HAR-1: electron density and geometry after HAR at the B3LYP/def2-TZVP level of theory without simulation of the environment.

2. HAR-CC-1: electron density calculated for the HAR-1 geometry at the same level of theory, but perturbed by a cluster of self-consistent point charges and dipoles surrounding the central molecule within a radius of 8 Å.

3. XCW-DFT/XCW-HF: electron density calculated using the XCW-fitting strategy at the B3LYP/def2-TZVP level of theory (DFT) and HF/def2-TZVP level of theory (HF) using the fixed HAR-1 geometry and fixed atomic displacement parameters (ADPs) determined also in HAR-1.

#### Strategy 2: variable geometry

Here, the geometry is optimized or refined for each model, allowing simultaneous variation of geometry, conformation, model of the electron density and polarization of the electron density in different environments. Consequently, both the dipole moment vector's distance parameter and the charges vary across the different models. This strategy allows us to explore the interdependence of these properties using QTAIM, analyzing parameters such as the electron density at bond critical points (BCPs), atomic charges, bond lengths, and dipole moments. Models in Strategy 2 are summarized below; more details are given in the section "Experimental details":

1. V-Opt: optimized geometry and related electron density of a single molecule in vacuum (V) at the B3LYP/def2-TZVP level of theory.

2. S-Opt: optimized geometry and related electron density in water solution at the B3LYP/def2-TZVP level of theory.

3. HAR-1: same as in Strategy 1.

4. HAR-CC-2: electron density and geometry after HAR at the B3LYP/def2-TZVP level of theory while the crystalline environment is simulated by a cluster of self-consistent point charges and dipoles.

Model HAR-1 is common to both strategies. The key distinction between HAR-CC-1 and HAR-CC-2 lies in their geometry: HAR-CC-1 uses a fixed geometry from HAR-1 with charges added afterwards, while in HAR-CC-2 the geometry was refined with cluster charges present.

### Experimental details

A small amount of commercially obtained *p*-arsanilic acid was added to a water–ethanol mixture (1 : 15 ratio) in a glass vial. The suspension was heated until it turned into a clear solution, then filtered to remove any insoluble particles. The filtered solution was sealed in the vial with parafilm, leaving two small holes for solvent evaporation. After two days, crystals formed.

A total of seven different low-temperature (100 K) single-crystal X-ray diffraction data sets were measured using both home-source and synchrotron radiation. At home, a Rigaku Synergy-S instrument with microfocus Cu radiation and a HyPix-6000HE detector as well as a Rigaku Synergy-R instrument with rotating anode Mo radiation and a HyPix-Arc 100° detector were used. Synchrotron experiments were carried out at the BL02B1 beamline of the SPring-8 synchrotron facility using a Dectris PILATUS-1M-CdTe pixel detector and a wavelength of 0.2483 Å (50 keV). Data reduction and absorption correction were done using the CrysAlisPro software. Crystal structures were solved with ShelxT^65^ and initially refined with ShelxL^66^ in the Independent Atom Model (IAM). A summary of these seven datasets with crystallographic, measurement and refinement details are provided in Tables S1 and S2 in the SI.

**Fig. 1:**
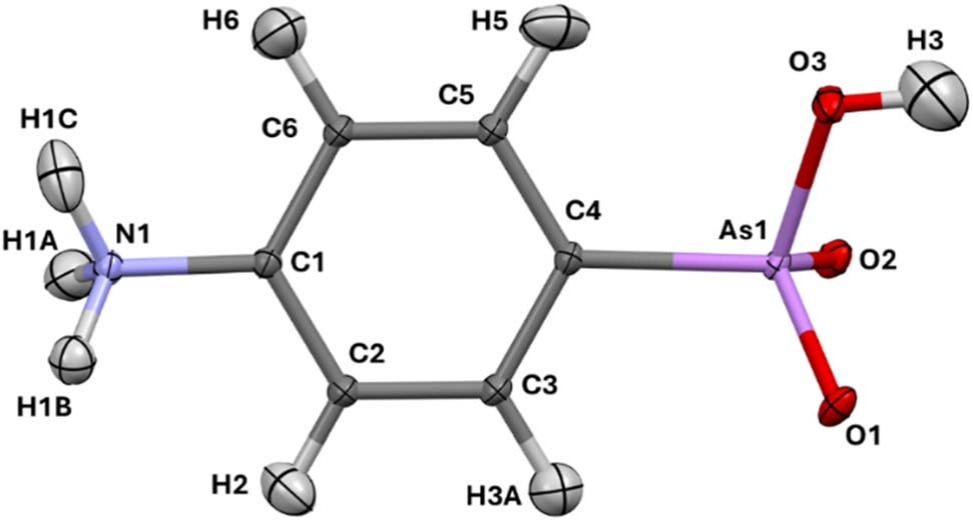
Molecular structure of *p*-arsanilic acid in its zwitterionic form based on HAR of dataset 5 with atomic labelling scheme. Hydrogen atoms were refined freely and anisotropically. ADPs shown at the 50% probability level, visualized using the Mercury software.^70^

To select the best dataset for building our models according to the two strategies discussed above, we applied a quantum crystallographic protocol (QCP) that was recently introduced by some of us*.*^67^ The QCP includes quantum crystallographic HARs using the NoSpherA2 software^68^ within Olex2.^69^ In the SI, we detail how we finally chose dataset 5 as the most suited one for further analysis. The refinement statistics are collected in Tables S3 and S4. The residual density isosurfaces and probability density function (PDF) isosurfaces are represented in Fig. S1 and S2. It is one of the synchrotron datasets (100 K, 50 keV), which is resolved up to *d* = 0.405 Å. Fig. 1 shows the corresponding molecular structure of *p*-arsanilic acid after HAR obtained from dataset 5 with freely refined hydrogen atom positions and ADPs as well as the atomic labeling scheme used throughout this study.

To maintain consistency across models, we performed X-ray wavefunction refinement (XWR),^71^ which combines Hirshfeld atom refinement (HAR) with X-ray constrained wavefunction (XCW) fitting, using the Tonto^72^ software. We first employed HAR of dataset 5 at the B3LYP/def2-TZVP level of theory without simulating the crystalline environment to generate model HAR-1. Based on this geometry and ADPs, we performed XCW fitting using Hartree-Fock (XCW-HF) with the def2-TZVP basis set and density functional theory (XCW-DFT) at the B3LYP/def2-TZVP level of theory, resulting in two additional models. Next, using the fixed geometry from HAR-1, we calculated a new wavefunction at the B3LYP/def2-TZVP level of theory perturbed by a surrounding self-consistent cluster of point charges and dipoles to create model HAR-CC-1.

For the second strategy, we further theoretically optimized the HAR-1 geometry, both in vacuum using the B3LYP/def2-TZVP level of theory (model V-Opt) and in water solution using the same level of theory (model S-Opt). The solvation model based on density (SMD) was employed to model the aqueous solution.^73^ The SMD is a continuum solvation model to simulate the effect of a solvent on a solute molecule. SMD is an implicit solvent model, meaning it represents the solvent as a continuous medium rather than using individual solvent molecules. It combines the polarizable continuum model (PCM) for electrostatic interactions with additional terms to account for non-electrostatic effects such as dispersion, cavitation, and solvent structure.^73^ For the geometry optimizations, the software Gaussian 09 (ref. 74) was used. Additionally, we performed HAR with a surrounding self-consistent cluster of point charges and dipoles in the software Tonto, fully refining the geometry to produce geometry and electron density of model HAR-CC-2. All computational details are provided in the SI. Statistical results for the quantum-crystallographic refinements HAR, XCW-HF, XCW-DFT, and HAR-CC-2 are summarized in Table S5.

A final geometry model of *p*-arsanilic acid (ARS140) was extracted from the literature:^75^ in that work, the interaction of *p*-arsanilic acid with a hen-egg-white lysozyme derivative was studied crystallographically, highlighting its potential as a phasing agent in protein crystallography and suggesting a strong binding affinity to protein residues.

## Results and discussions

### Crystal packing and intermolecular interactions

**Fig. 2:**
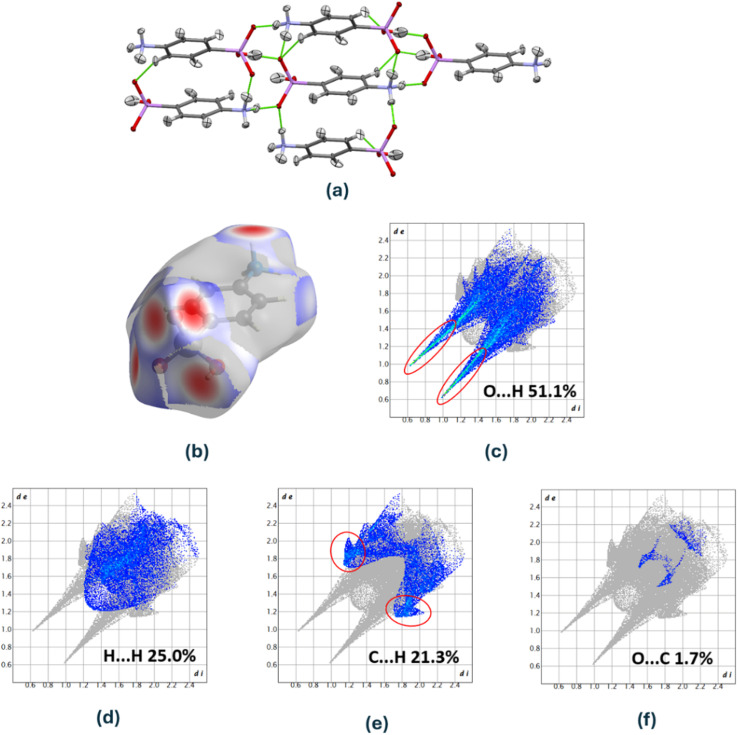
(a) Hydrogen bonds and other interactions in the crystal packing of *p*-arsanilic acid. White, gray, blue, red, and purple atoms represent hydrogen, carbon, nitrogen, oxygen, and arsenic atoms, respectively. Hydrogen bonds are shown as green dashed lines between a central *p*-arsanilic acid molecule (HAR-1 model) and its closest symmetry-generated neighbours in the crystal packing. (b) Hirshfeld surface of *p*-arsanilic acid with the property *d*_norm_ mapped onto it, only for those regions that represent O⋯H contacts (51.1% of the total surface area). Red color represents O⋯H contacts for which the contact distance mediated by the surface is smaller than the sum of the O and H van-der-Waals radii. (c) Hirshfeld surface fingerprint plot, only for those surface points that represent O⋯H contacts. The sharp spikes encircled in red represent the hydrogen bonds. (d) Fingerprint plot breakdown for H⋯H contacts. (e) Fingerprint plot breakdown for C⋯H contacts. Regions of C–H⋯π interactions with the typical chicken-wing like features are encircled in red. (f) Fingerprint plot breakdown for C⋯O contacts. The software CrystalExplorer was used.^79^

We have previously investigated a broad range of intermolecular interactions involving arsenic atoms that can serve as Lewis acid or base depending on the oxidation state.^76^ Here, the As atom is not involved in intermolecular contacts; the crystal packing of *p*-arsanilic acid is governed by many strong O–H⋯O and N–H⋯O hydrogen bonds. Thus, the melting point of *p*-arsanilic acid is over 300 °C. Fig. 2(a) shows that all ammonium N–H and the arsenite O–H donor groups interact with the two partially negatively charged oxygen atoms of the arsenite group as acceptors, forming patterns of bi- and tri-furcated hydrogen bonds. Some of the C–H bonds in the phenyl ring are also involved with the same arsenite acceptor oxygen atoms in weaker C–H⋯O hydrogen bonds. The complete list of hydrogen bonds is given in Table S6. In addition, Fig. S3 and Table S6 show a network of intermolecular CH⋯π interactions and close contacts between the hydroxyl oxygen atoms and the phenyl rings.

We further performed Hirshfeld surface analysis^77^ for *p*-arsanilic acid to shed more light on the distribution of such types of interactions. Fig. 2(b) shows the Hirshfeld surface with those 50% of the surface color coded that are covered with O⋯H contacts, of which the areas in red color represent the hydrogen bonds. They are also depicted with sharp spikes in the fingerprint plot^78^ in Fig. 2(c). Further 21% of the surface are C–H⋯C contacts. The chicken-wing features in the corresponding fingerprint plot (Fig. 2(e), circled in red) represent those contact pairs that are attractive C–H⋯π interactions. There are also some interesting O⋯C contacts (only 1.7% of the overall surface, Fig. 2(f)) of which the closest ones may be interpreted as lone-pair⋯π interactions. The remaining 25% belong to H⋯H contacts, some of which represent attractive London dispersion interactions as shown by the central broad spike in the fingerprint plot of Fig. 2(d). Overall, there is a variety of strong and attractive interactions in the crystal packing of *p*-arsanilic acid that can all be leveraged in the biological environment or in solution, too. It will be interesting to analyze how these interactions polarize the central molecule and how they change the geometry of the molecule in different environments to maximize the attractive interactions. The other Hirshfeld surfaces and fingerprint plots are represented in Fig. S4 and S5.

### Different geometries and chirality

**Fig. 3:**
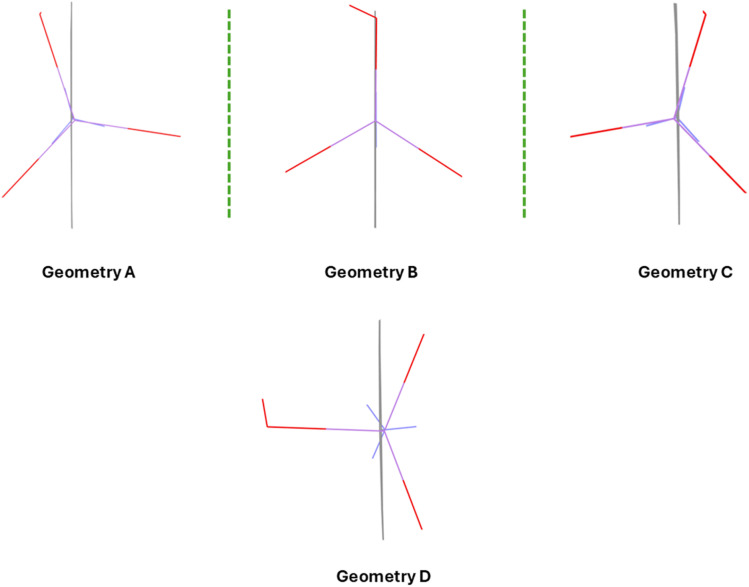
Different geometries adopted by *p*-arsanilic acid in different environments. (A) Single crystal, first enantiomer, (B) co-crystal with lysozyme protein, (C) single crystal, second enantiomer, (D) DFT-optimized in vacuum. Geometry A is from the crystal structure of dataset 5, while geometry C is obtained from dataset 1.

We consider three different geometries of *p*-arsanilic acid as study case: (i) geometry obtained from DFT optimization in the vacuum for isolated *p*-arsanilic acid (model V-Opt); (ii) experimental geometry resulting from HAR refinement (HAR-1); (iii) experimental geometry of *p*-arsanilic acid while binding to a lysozyme protein. Geometries (i) and (ii) were obtained by us, but conformation (iii) was extracted from the protein crystal structure with PDB code 1N4F.^75^ In the co-crystal with lysozyme, there are three independent *p*-arsanilic acid ligands which are bonded to protein residues (see Fig S6, and Tables S7–S9). We have chosen one of them (residue ARS140) for further analysis as the geometrical parameters are similar among the three, especially concerning the conformation caused by rotation of the arsenite (AsO_3_) group. When comparing the optimized geometry, the single-crystal HAR geometry and that of ARS140, the conformations caused by rotation of the arsenite group are, in turn, quite different (Fig. 3) although the bond lengths and angles differ only slightly. Hence, we attribute these conformational changes to the fact that the molecule adapts the positions of the hydrogen bond donor and acceptor groups to maximize interactions with the environment.

In fact, Fig. 3 represents not three but four different conformations of *p*-arsanilic acid. In the single-crystal structure, *p*-arsanilic acid adopts either geometry A or C. These two geometries are mirror images of each other; therefore, the two related molecules are enantiomers of each other as long as the rotation is hindered as it is in the crystal packing. This is a case of planar chirality with respect to the phenyl ring as the molecular plane (depicted as a gray line in Fig. 3). The relationship between this molecular chirality and the helical crystal packing in the Sohncke space group *P*2_1_ is not trivial. We refer to a similar discussion of molecular planar chirality *vs.* helical crystal packing found for the YLID test crystal.^80^ In any case, we find spontaneous resolution into two enantiomerically pure crystal forms when crystallizing *p*-arsanilic acid that can be distinguished from each other unambiguously by their anomalous dispersion signal (Table S1 for refined Flack/Hooft parameters). The hydroxyl group is rotated away from the molecular plane either to the left or to the right. Geometry B acts like a transition state between A and C. This is the geometry that *p*-arsanilic acid adopts in the crystal structure with lysozyme; here, the hydroxyl group is in the molecular plane. In the optimized geometry (geometry D), the hydroxyl group has turned away from the molecular plane to a maximum extent (90° to the molecular plane).

**Fig. 4:**
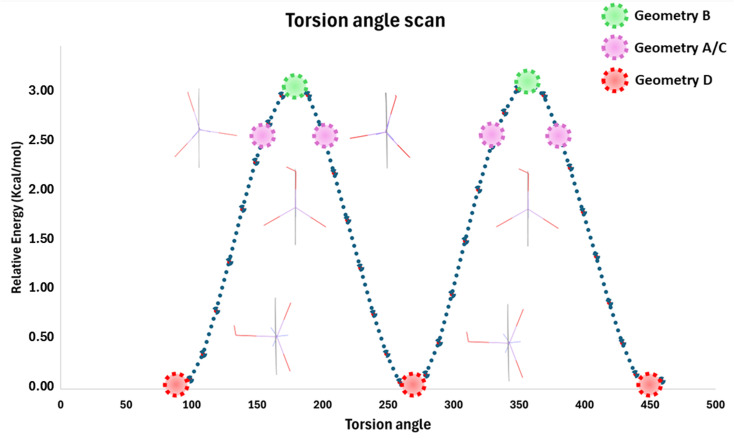
Relaxed potential energy surface scan of *p*-arsanilic acid by changing the O_3_–As_1_–C_4_–C_5_ torsion angle by 10° per step. This represents the rotation of the arsenite group relative to the molecular plane defined by the phenyl ring. The scan was done at the B3LYP/def2TZVP level to obtain the different geometries. Then, the energies were calculated with the CCSD method on fixed DFT-geometries.

Intermolecular interactions with the environment are obviously energetically large enough to overcompensate the barrier of rotation and to produce various conformers. This flexibility of the arsenite (AsO_3_) group to adapt itself to the environment motivated us to calculate the rotational barrier to have a better perspective about the relationships between these geometries. We performed a relaxed scan of the potential energy surface (PES) at the B3LYP/def2-TZVP level of theory by changing the O_3_–As_1_–C_4_–C_5_ torsion angle; 37 steps with a step size of 10°. Fig. 4 represents our result for the relaxed PES scan. When O_3_–As_1_–C_4_–C_5_ equals 90°/270°, the energy of the system is at its minimum (geometry D). On the other hand, when the angle is 0°/180°/360°, the energy of the system is maximum, which represents the planar geometry in the protein environment (geometry B). In the case of the single-crystal structures, the O_3_–As_1_–C_4_–C_5_ torsion angle is about 20° to either side of the molecular plane, which is energetically closer to the maximum of the PES scan than the minimum.

Since the standard deviation on the energies of DFT calculations is in the range of 2–5 kcal mol^−1^,^81^ which is in the same range as the energy of hydrogen bonds, we employed coupled-cluster calculations with singlet and doublet excitations (CCSD) at the fixed geometries from the DFT PES scan for better relative energies. The rotation barrier, which means the energy difference between the vacuum-optimized geometry D and the geometry in the protein environment (geometry B), is 3.00 kcal mol^−1^ (12.57 kJ mol^−1^). The difference between geometry D and geometry A/C is 2.63 kcal mol^−1^ (11.01 kJ mol^−1^). These energy barriers are clearly below 5 kcal mol^−1^, which is an approximate value that different environments can provide at room temperature.^82,83^ This confirms that the energy provided by the intermolecular interactions that were discussed in the previous section allows the *p*-arsanilic acid molecule to easily adapt to different environments. It is curious, though, that in the biological environment all three independent molecules are locked into planar conformations, which is the transition state of the rotation in the vacuum. This may be a bias in the protein crystal structure due to low accuracy, but it may also be an effect of the biological environment. For more conclusions regarding the recognition of the small molecule (*p*-arsanilic acid) in the protein, we need to consider the polarization of the electron density and the related electrostatic potential.

### Interaction density and interaction ESP

We defined the following models in Strategy 1 – fixed geometry in the experimental and computational section: HAR-1, HAR-CC-1, XCW-DFT, and XCW-HF. Interaction densities and interaction electrostatic potentials are calculated based on these models. The interaction densities and interaction ESPs are obtained by subtracting the electron density and ESP grids of model HAR-1 from those of the models HAR-CC-1, XCW-DFT, and XCW-HF, respectively. The result of the subtraction is a grid file that includes the difference between electron density (ED) or ESP at each grid point. This aids us to visualize the interaction ED or interaction ESP, respectively, always in the definition of two models. In this strategy, the geometry of *p*-arsanilic acid is fixed and the same among different models. Therefore, changes in the electron-density distributions are purely due to polarization, not mediated by geometry changes. If we consider the absolute values for ED and ESP from the grids, the differences that represent the impact of polarization are small. For example, the maximum ED value from the original grid of model HAR-1 is 314 e Å^−3^ while the maximum of ED in the interaction density grid (HAR-CC-1 minus model HAR-1) is 0.75 e Å^−3^. When comparing the isosurface values chosen for the visualization in our interaction density plot (±0.009 a.u. = ±0.061 e Å^−3^) to those reported for reference calculations in ref. 48 (Fig. 7, ±0.0025 a.u. = ±0.017 e Å^−3^), the effect of polarization is slightly smaller in the case of *p*-arsanilic acid than in the organic reference compounds urea and l-alanine, because the values were chosen to represent isosurfaces of approximately the same spatial extension.

**Fig. 5:**
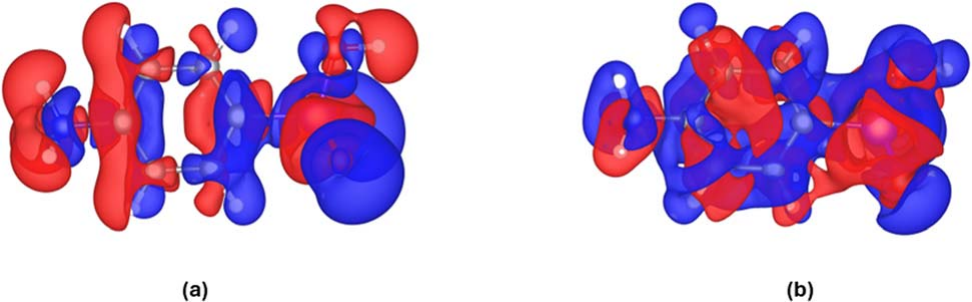
Interaction densities for (a) model HAR-CC-1 minus model HAR-1, and (b) model XCW-DFT minus model HAR-1. Blue = positive, red = negative. The isosurface value is ± 0.009 a.u. = ± 0.061 e Å^−3^.

In the interaction density maps in Fig. 5, the red region represents negative parts and the blue regions are positive parts. This means that blue regions indicate electron gain due to the simulation of the crystal field, whereas red regions indicate electron loss. A purely electrostatic estimate of the polarization is given by Fig. 5(a) as it depicts the perturbation of the electron density by a cluster of point charges and dipoles. The polarization effect spans the entire molecule, and is not restricted to those atoms in strong intermolecular interactions. The electronegative oxygen and nitrogen atoms accumulate electron density in the crystal field, whereas their hydrogen atoms lose electron density, which is consistent with the idea of a hydrogen-bonded interaction to be mainly of electrostatic nature. The As atom is enclosed by a large region of negative interaction density, meaning that the charge separation between As and O atoms is increased by the crystal field. Interestingly, the direction of polarization of the π-electron cloud of the phenyl ring is reversed between the two halves of the ring.

The discussed polarization effect based on cluster charges is a purely theoretical simulation. Experimentally, both polarization and electron correlation can be estimated *via* X-ray constrained wavefunction fitting.^48,84^ Hence, both models XCW-HF and XCW-DFT include both physical effects. In fact, it was shown that electron correlation builds up in the HF wavefunction *via* fitting but will be underrepresented even at high values of the perturbation multiplier λ.^32,48^ In the DFT wavefunction, electron correlation is strongly overrepresented and this overestimate will be reduced *via* XCW fitting, but not completely even at high *λ* values. So, in fact, an average of the electron densities of XCW-HF and XCW-DFT should be the best estimate of the experimentally fitted electron correlation effect. We have produced this average XCW-HF/DFT electron density here (Fig. S7), and we believe that it could be a good starting point for benchmarking DFT functionals, compare ref. 84 However, this is out of scope for this investigation.

Here, we study the difference electron density between XCW-DFT and HAR-1 (Fig. 5(b)). This is an experimental estimate of polarization plus some remainders of electron correlation that are not part of the B3LYP ansatz, so the resulting map should somehow resemble the interaction density. The similarity between Fig. 5a and b is relatively low, although some main features such as the strong negative region around the As atom as well as the direction of polarization of the phenyl ring in two halves are preserved.

**Fig. 6:**
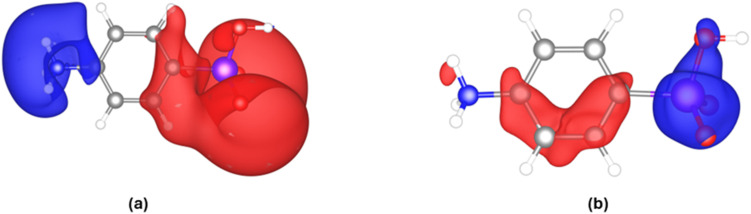
Interaction ESPs for (a) model HAR-CC-1 minus model HAR-1, and (b) model XCW-DFT minus model HAR-1. Blue = positive, red = negative. The isosurface value is ± 0.3 a.u. = ± 0.56 e Å^−1^ = 8.2 V. As atom bonded to C4 (see labelling scheme in Fig. 1).

In the interaction ESP plot in Fig. 6(a), the overall increase of the polarization of the zwitterionic molecule becomes clear. The positive ammonium side of the molecule becomes even more positive, and the negative arsenite side more negative. This increased polarization is expected in an electrostatic crystal field. An analysis of the QTAIM charges across different models reveals (absolute values in the SI) that the atoms exhibiting the greatest fluctuations in their atomic charges are arsenic, all oxygen atoms, nitrogen, and C4, which is bonded to As. In contrast, the remaining atoms show relatively consistent charge values across models. For instance, in model HAR-1, the charges on As and C4 are +2.60e and −0.31e, respectively. In comparison, in model HAR-CC-1 these charges become slightly higher upon polarization (+2.65e for As, −0.41e for C4; the distribution of all QTAIM charges for this strategy is plotted in Fig. S8). C4 becomes more negative, so a red surface around it in Fig. 6(a) is consequential. The small positive increase at the As atom is not reflected by a blue isosurface in Fig. 6(a) at the chosen isovalue.

However, this effect is much more pronounced in the XCW-DFT minus HAR-1 map (Fig. 6(b)). Here, the increase of positive charge at the As atom (blue surface) dominates the entire arsenite functional group including its oxygen atoms and is accompanied by an increased negative charge of the phenyl ring (red isosurface). This means that the As–C4 bond becomes more polar, and this is the main effect that was fitted from the experimental structure factors. This is reflected in the QTAIM charges: In model HAR-1, the charges are As = +2.60e and C4 = −0.31e, which become much more extreme in model XCW-DFT with As = +2.95e and C4 = −0.65e. The charge difference between these two atoms (the bond polarity) increases by Δ*q* = 0.70 e, whereas between models HAR-1 and HAR-CC-1 the increase in charge separation is only Δ*q* = 0.15 e. This shows that the purely electrostatic theoretical estimation of the crystal field effect by cluster charges is insufficient (compare ref. 85); here, fitting to experimental structure factors provides a much stronger polarization of the As–C4 bond. Overall, the As–C4 bond is the one that is affected most strongly by polarization, although its atoms are not directly involved in intermolecular interactions. This means that the impact of the As atom on the polarization of the entire *p*-arsanilic acid zwitterion needs further clarification.

### Dipole moments and flexible geometries

**Fig. 7:**
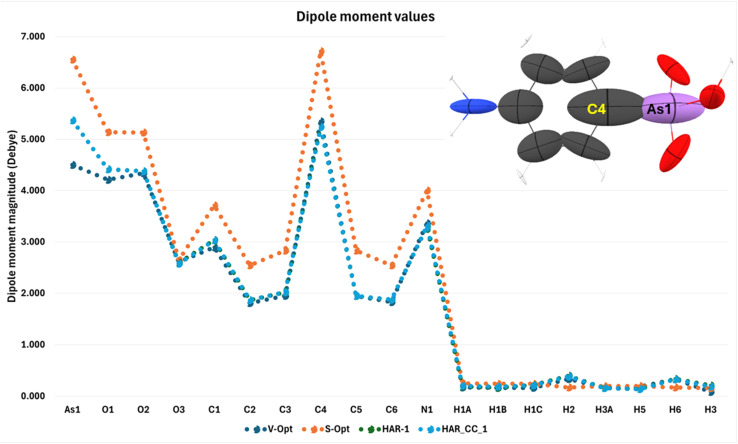
Magnitude of the atomic dipole moments in four different environments. The picture on the top right represents the polarizabilities calculated using PolaBer.^55^

Following our second strategy (Experimental and Computational Section), we have defined the following models to examine the changes of conformation, polarization and electron-density distribution at the same time: V-Opt, S-Opt, HAR-1, HAR-CC-2. Apart from bond distances, we study the following QTAIM parameters: electron density values at BCPs (bond critical points), atomic charges, and atomic dipole moments (Table S10 and Fig. S9). Since the QTAIM approach employs a partitioning scheme, the total dipole moment of the molecule is constructed from the sum of atomic dipoles. Each atomic dipole has two components: atomic polarization and charge translation. By integrating the dipolar density function within the atomic basins, atomic polarization is built which is the interatomic contribution. The charge translation component depends on the nature and the number of coordinating groups to that atom and contains information about “bond charges”. To calculate these parameters, we employed QTAIM analysis within the software AIMALL^86^ and here we focus only on the total atomic dipole moments. According to Fig. 7, there is no difference in the two vacuum models V-Opt and HAR-1 although they are based on different geometries (theoretically optimized *versus* experimentally refined). This means that it is the actual perturbation of the electron density by the different environments that is responsible for the internal polarization. The atomic dipole moments are different especially between the solvation model and the others. For the As atom, the simulation of the crystal field by cluster charges also gives rise to a significant increase in the atomic dipole moment. Overall, it is especially the As1–C4 bond that seems to be most affected by the environment (compare with Fig. 6(b), and discussion around it).

A calculation of the atomic polarizabilities (inlet in Fig. 7) shows that these two atoms clearly have the largest polarizabilities, which governs the intramolecular polarization of the molecule induced by the intermolecular interactions. The software PolaBer uses QTAIM partitioning to define the electron density basins for individual atoms. To compute atomic polarizability, six different wavefunctions are required—each corresponding to the application of an electric field in a different direction: +X, −X, +Y, −Y, +Z, and −Z. PolaBer analyzes all six wavefunctions to evaluate how the electron density within each atomic basin responds to these external fields. The ellipsoids shown in Fig. 7 illustrate both the magnitude and direction of the electron density's polarization within each basin. In simple terms, a larger ellipsoid indicates a greater capacity for electron density distortion, reflecting higher polarizability. Here, the biggest differences are for atoms As1 and C4. This sheds some light on the specific role of arsenic in this biologically active molecule.

## Conclusions

The biologically active *p*-arsanilic acid and its derivative atoxyl have a rich history of use and abuse, which must not be forgotten, and which is therefore summarized in this paper. The main scientific interest is the role of the arsenic atom on the biological activity. Our quantum crystallographic analysis shows that the As atom is not involved directly in any intermolecular interactions that are normally understood as being most important for the biological recognition process. Instead, we show that the As atom has the highest atomic polarizability together with the bonded carbon atom C4, which in turn leads to this bond being most polarized in the environment. This means that the inherently large polarizability of the As atom is responsible for the overall molecular response of the drug molecule to its environment, which is induced by intermolecular interactions. The environment is here represented by the crystal field and a solvation model. We assume that the same conclusion is true for the biological environment, as it has been shown previously that the small-molecule crystal structure is a very good approximation of the bonding situation in an enzyme.^30–35,37^ Therefore, we speculate that the large inherent polarizability and effective polarization of the arsenic-carbon bond is one of the reasons for atoxyl to be that toxic.

The method X-ray constrained wavefunction fitting embeds experimental diffraction data into a molecular wavefunction calculation. We find in this study that this method most reliably detects the polarization of the As–C bond; a theoretical simulation of the crystal field effect is not sufficient, and a theoretical simulation of the solvent effect overrepresents the effect. Experimental data are important to find the correct order of magnitude of the interaction density, the interaction electrostatic potential, and the related atomic charges.

We observed spontaneous enantiomeric resolution of *p*-arsanilic acid in the solid state, which is not chiral in solution or the gas phase. The absolute configurations of both enantiomers can unambiguously be elucidated by the anomalous signal in the X-ray diffraction experiment. The chirality of the compound in the solid state was not discussed before, and its absolute configuration was not given, although the crystal structure was obtained before.^87^ The Na salt atoxyl is not chiral in its crystal structure.^88^ Hence, we do not have any evidence whether the chirality of zwitterionic *p*-arsanilic acid, which is generated by strong interactions with the environment, has an effect on the biological activity. However, we would like to draw attention to the possibility of planar chirality of this important compound.

## Conflicts of interest

There is no conflict of interest to declare.

## Supplementary Material

RA-015-D5RA05936D-s001

RA-015-D5RA05936D-s002

## Data Availability

CCDC 2465705, 2465706, 2465707, 2465709, 2465822, 2465823, and 2465824 contain the supplementary crystallographic data for this paper as crystallographic information files.^89*a*–*g*^ Further supplementary information is available as a pdf document. See DOI: https://doi.org/10.1039/d5ra05936d.
