# Supplementary material for: Interplay between geometry, electron density, and polarizability of the controversial drug atoxyl in crystal and biological environments
Source: RSC Adv. 2025 Nov 27;15(54):46749–60. doi: 10.1039/d5ra05936d (PMC12658475; doi:10.1039/d5ra05936d)
Supplement: RA-015-D5RA05936D-s001 [file RA-015-D5RA05936D-s001.pdf]

Supplementary Information (SI) for RSC Advances.
This journal is © The Royal Society of Chemistry 2025

Supplementary Information

Interplay between geometry, electron density, and polarizability of the controversial drug atoxyl in crystal and biological environments

Yaser Balmohammadi[1], Eduardo Metry[1], Lorraine A. Malaspina[1], Georgia Cametti[2], Yuiga Nakamura[3], Simon Grabowsky[1]*

[1] University of Bern, Department of Chemistry, Biochemistry and Pharmaceutical Sciences, Freiestrasse 3, 3012 Bern, Switzerland

[2]University of Bern, Institute of Geological Sciences, Baltzerstrasse 3, 3012 Bern, Switzerland

[3]Japan Synchrotron Radiation Research Institute (JASRI), Sayo-cho, Hyogo 679-5198, Japan

* Correspondence e-mail: simon.grabowsky@unibe.ch

We collected seven single-crystal X-ray diffraction datasets of p-arsanilic acid at 100 K using both home-source and synchrotron radiation. Details about the instrumentation and the measurements are given in the main text (Experimental and Computational Section, part III). Crystallographic and measurement details are provided in Tables S1 and S2 below. We followed the quantum crystallographic protocol (QCP) that we introduced earlier this year.[1] To follow the steps of QCP, first, we refined the structures using the independent atom model (IAM), followed by HAR using NospherA2. Tables S3 and S4 summarize the statistical data for these refinements. It should be noted that anharmonic motions of the arsenic atom were refined using Olex2 for all datasets, except for datasets 2 and 7. The anharmonic refinements were performed due to the observation of a residual density pattern resembling a "shashlik" for the arsenic atoms. Further details, including an evaluation of the probability density function (PDF) isosurfaces, are provided in Figure S1 and the related text.

We needed to select the best dataset among the seven data sets of p-arsanilic acid to be taken forward for the chemical discussion in the main article. Dataset 3 exhibits characteristics of a twin crystal and was therefore excluded from further analysis. Datasets 2 and 7, which exhibit lower resolutions (0.827 Å and 0.557Å, respectively) relative to the other datasets, were also set aside, as higher resolution datasets are preferred for conducting experimental electron-density analysis. Among the remaining datasets, datasets 6 and 1 display higher residual electron densities following HAR (Table S4) and lower resolution (Table S1) when compared to datasets 4 and 5. Additionally, despite undergoing anharmonic refinement, dataset 1

continues to show significant residual electron density around the arsenic atom (Figure S2). Datasets 4 and 5 are closely comparable; however, subtle differences distinguish them. The residual electron density in dataset 5 (+0.291/-0.260 e/Å^3) is more balanced than in dataset 4 (+0.214/-0.277 e/Å^3). Besides residual density, the anisotropic displacement parameters (ADPs) of hydrogen atoms after HAR in dataset 5 adopt more appropriate shapes, with ellipsoids elongating perpendicularly to the bond direction. By contrast, in dataset 4, four hydrogen atoms required ISOR restraints due to the flattened and unsuitable shapes of their ADPs, a limitation that is not observed in dataset 5. Consequently, dataset 5 was identified as the optimal dataset for subsequent analysis.

Table S1. Single crystal X-ray diffraction datasets for *p*-arsanilic acid

Dataset number	Max. resolution (Å)	Wavelength (Å)	Crystal	Enantiomer	Flack/Hooft parameter[&]
Dataset 1	0.452	Mo= 0.71073	Crystal 1	Enantiomer C	0.0013(16)
Dataset 2	0.827	Cu= 1.54184	Crystal 6	Enantiomer A	-0.014(7)
Dataset 3[#]	0.408	synchrotron = 0.2483	Crystal 2	Enantiomer C	-0.008(8)
Dataset 4	0.408	synchrotron = 0.2483	Crystal 2[$]	Enantiomer C	-0.013(8)
Dataset 5	0.405	synchrotron = 0.2483	Crystal 3	Enantiomer A	0.001(9)
Dataset 6	0.474	synchrotron = 0.2483	Crystal 4	Enantiomer A	-0.012(19)
Dataset 7	0.557	synchrotron = 0.2483	Crystal 5	Enantiomer A	-0.05(4)

*All the datasets were collected at 100K.

This dataset was treated as a twin.

$ The crystal was reshaped to a more spherical form by dissolving its edges before it was remeasured under the same conditions.

& The Flack parameter values are calculated based on the Hooft variant: R. W. W. Hooft, L. H. Straver, A. L. Spek, *J. Appl. Cryst.*, **2010**, *43*, 665-668.

Table S2. Crystallographic and measurement details for all datasets

Dataset	Dataset 1	Dataset 2	Dataset 3
**Chemical formula**	$C_6H_8NAsO_3$	$C_6H_8NAsO_3$	$C_6H_8NAsO_3$
**Form. weight (g/mol)**	217.06	217.06	217.06
**Crystal size (mm)**	0.26 0.15 0.05	0.17 0.13 0.09	0.12 0.11 0.10
**Crystal habit**	block	block	block
**Crystal color**	colourless	colourless	colourless
**Temperature (K)**	100	100	100
**Wavelength (Å)**	0.71073	1.54184	0.2483
**a (Å)**	7.23467(5)	7.23374(4)	7.24136(5)
**b (Å)**	6.13615(4)	6.13187(2)	6.13653(4)
**c (Å)**	8.69951(5)	8.69579(5)	8.70452(6)
**α (°)**	90	90.0	90.0
**β (°)**	101.0397(6)	101.0942(5)	101.0741(7)
**γ (°)**	90	90.0	90.0
**Volume (Å^3)**	379.051(4)	378.505(3)	379.599(4)
**Z, Z'**	2,1	2,1	2,1
**Space group**	$P\ 1\ 2_1\ 1$	$P\ 1\ 2_1\ 1$	$P\ 1\ 2_1\ 1$
**Number of refl.**	156885	46129	116313
**Rint/Compl./Red.**	3.99%/99.7%/19.83	2.22%/99.9%/32.90	5.86%/99.9%/9.96
**Unique reflections (used in ref.)**	7911	1402	11676
**Unique reflections with I>2σ(I)**	7620	1401	11232
**Reflns theta min°**	3.36	5.18	1.53
**Reflns theta max°**	49.84	68.65	17.71
**Resolution (Å)**	0.465	0.827	0.408
**I/σ(I)**	65.5	200.0	40.3
**CCDC Number**	2465709	2465706	2465822
**Dataset**	Dataset 4	Dataset 5	Dataset 6
**Chemical formula**	$C_6H_8NAsO_3$	$C_6H_8NAsO_3$	$C_6H_8NAsO_3$
**Form. weight (g/mol)**	217.06	217.06	217.06
**Crystal size (mm)**	0.06 0.05 0.035	0.10 0.085 0.08	0.18 0.14 0.10
**Crystal habit**	block	block	block
**Crystal color**	colourless	colourless	colourless
**Temperature (K)**	100	100	100
**Wavelength (Å)**	0.2483	0.2483	0.2483
**a (Å)**	7.24314(5)	7.24395(6)	7.24300(8)
**b (Å)**	6.13919(4)	6.13599(4)	6.13597(7)
**c (Å)**	8.70641(6)	8.70666(6)	8.70648(9)
**α (°)**	90.0	90.0	90.0
**β (°)**	101.0748(5)	101.0709(6)	101.0696(9)
**γ (°)**	90.0	90.0	90.0
**Volume (Å^3)**	379.938(4)	379.799(5)	379.741(7)
**Z, Z'**	2,1	2,1	2,1

**Space group**	$P\ 1\ 2_1\ 1$	$P\ 1\ 2_1\ 1$	$P\ 1\ 2_1\ 1$
**Number of refl.**	116568	117366	71388
**Rint/Compl./Red.**	5.14%/98.9%/10.05	4.95%/98.9%/9.80	5.95%/99.1%/9.59
**Unique reflections (used in ref.)**	11687	11979	7450
**Unique reflections with I>2σ(I)**	11596	11904	7348
**Reflns theta min°**	1.53	1.53	1.53
**Reflns theta max°**	17.71	17.85	15.18
**Resolution (Å)**	0.408	0.405	0.474
**I/σ(I)**	52.4	51.6	37.0
**CCDC Number**	2465705	2465823	2465824

**Dataset**	Dataset 7
**Chemical formula**	$C_6H_8NAsO_3$
**Form. weight (g/mol)**	217.06
**Crystal size (mm)**	0.15 0.10 0.09
**Crystal habit**	block
**Crystal color**	colourless
**Temperature (K)**	100
**Wavelength (Å)**	0.2483
**a (Å)**	7.24488(14)
**b (Å)**	6.14439(11)
**c (Å)**	8.70530(17)
**α (°)**	90.0
**β (°)**	101.1065(16)
**γ (°)**	90.0
**Volume (Å^3)**	380.261(12)
**Z, Z’**	2,1
**Space group**	$P\ 1\ 2_1\ 1$
**Number of refl.**	138701
**Rint/Compl./Red.**	7.88%/99.3%/30.23
**Unique reflections (used in ref.)**	4596
**Unique reflections with I>2σ(I)**	4407
**Reflns theta min°**	1.53
**Reflns theta max°**	12.88
**Resolution (Å)**	0.557
**I/σ(I)**	19.4
**CCDC Number**	2465707

Table S3. Refinement statistics for all datasets- IAM

	Dataset 1	Dataset 2	Dataset 3	Dataset 4	Dataset 5	Dataset 6	Dataset 7
**Number of parameters**	132	133*	132	132	132	133*	132
**R factor (%, I>2σ(I))**	1.17	1.05	1.56	1.18	1.25	1.60	1.97
**wR factor (%)**	2.77	2.74	3.58	2.90	3.02	3.75	5.18
**Goodness of fit**	1.02	1.14	1.00	1.05	1.05	1.04	0.92
**Residual density max (e/Å^3)**	+0.490	+0.234	+0.665	+0.510	+0.382	+0.589	+0.491
**Residual density min (e/Å^3)**	-0.190	-0.183	-0.491	-0.278	-0.650	-0.330	-0.369

* The extra parameter indicates the refinement of extinction in these datasets.

Table S4. Refinement statistics for all datasets- HAR using NoSpherA2

	Dataset 1	Dataset 2	Dataset 3	Dataset 4	Dataset 5	Dataset 6	Dataset 7
**Number of parameters***	197	167	192	197	198	196	162
**R factor, (%, I>2σ(I))**	0.94	0.69	1.44	0.87	0.86	1.09	1.27
**wR factor (%)**	1.99	1.64	3.25	2.05	2.00	2.33	3.31
**Goodness of fit**	0.83	1.24	0.99	1.39	1.07	0.99	0.83
**Residual density max (e/Å^3)**	+0.294	+0.120	+0.353	+0.206	+0.216	+0.323	+0.215
**Residual density min (e/Å^3)**	-0.214	-0.163	-0.382	-0.250	-0.254	-0.223	-0.277

* The numbers of parameters are different because of the refinement of Gram-Charlier parameters in all but 2 and 7, as well as the use of restraints and constraints on hydrogen atoms if needed.

Refinement of anharmonic motion

Except for datasets 2 and 7, a residual density pattern resembling a "shashlik" was observed for the arsenic atom. Consequently, Gram-Charlier parameters describing the anharmonic atomic motions were refined for the arsenic atoms during the HAR using NoSpherA2. This refinement resulted in a reduction in the minimum and maximum values of the residual density, an improvement in the R factors of the refinement, and the elimination of the shashlik-like residual density pattern. To assess the physical validity of refining anharmonic motions, we examined the total probability density function (PDF) isosurfaces (Figure S1) along with the Cijk and Dijkl values (Gram-Charlier parameters, provided in the deposited CIFs) from the refinements. According to the isosurfaces, the negative regions are minimal and located at the periphery

of the PDF domains, never within the core. For each anharmonic refinement, certain Cijk and Dijkl values were clearly significant, exceeding three times the standard uncertainty.

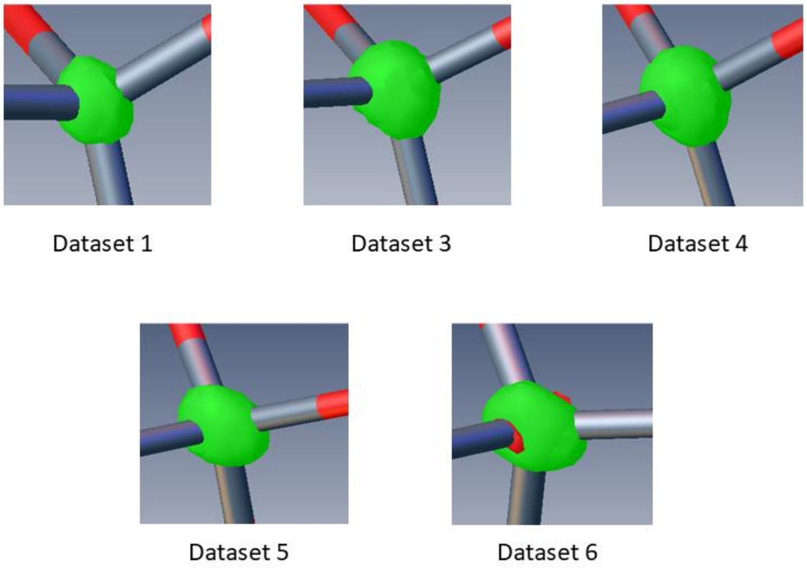

Figure S1. Total PDF (probability density function, 2nd, 3rd and 4th order) for the arsenic atom. Green = positive, red = negative. The images are generated using the software Olex2.

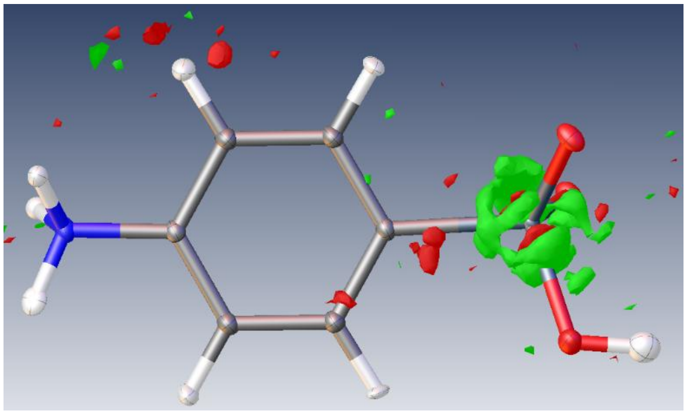

Dataset 1

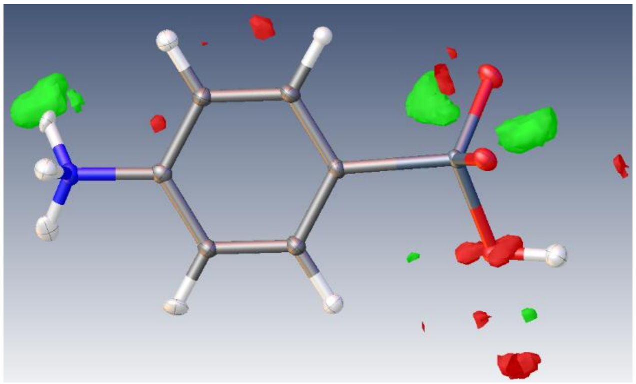

Dataset 2

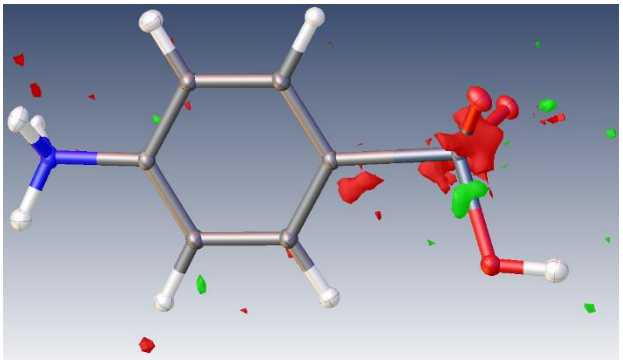

Dataset 3

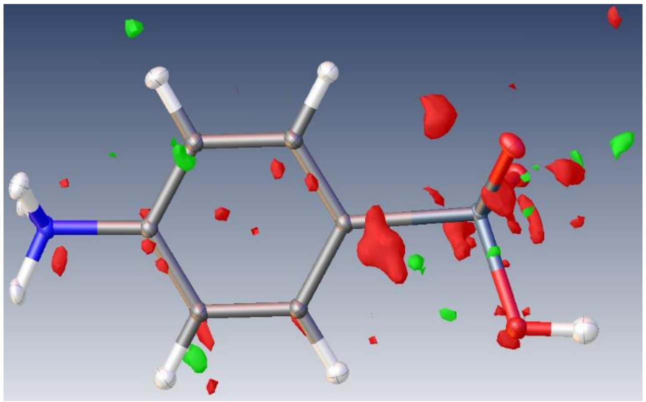

Dataset 4

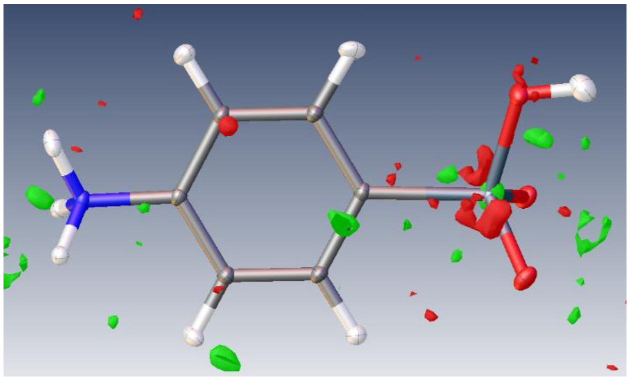

Dataset 5

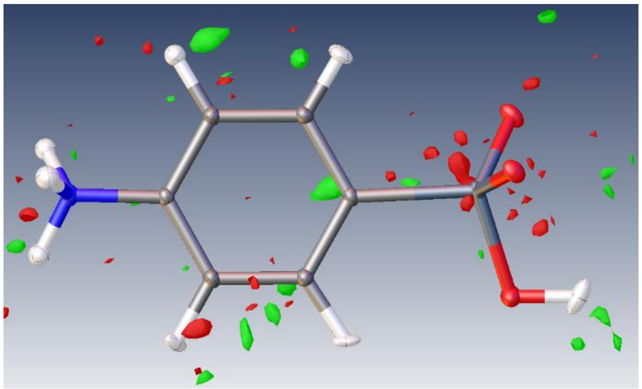

Dataset 6

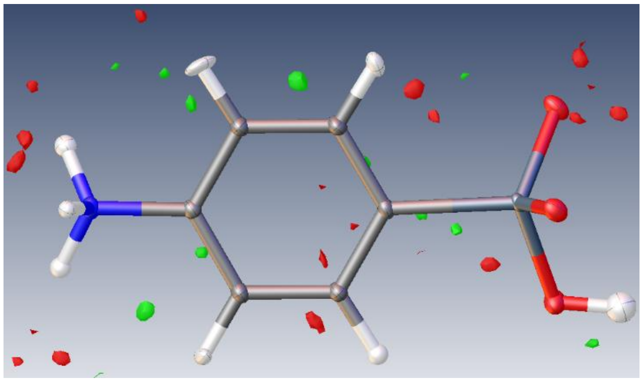

Dataset 7

Figure S2. Residual electron density isosurfaces for all datasets after HAR. Green = positive, red = negative. Isosurface values are ±0.1 e/Å^3. The images were generated using the Olex2 software.

Table S5. Refinement statistics of XWR for dataset 5 using the software Tonto

	HAR	HAR_CC*	XCW with DFT	XCW with HF
**R factor**	0.010	0.009	0.008	0.009
**wR factor**	0.010	0.010	0.009	0.010
**Goodness of fit**	1.268	1.242	1.148	1.239
**Residual density max (e/Å^3)**	0.173	0.168	0.159	0.163
**Residual density min (e/Å^3)**	-0.317	-0.299	-0.265	-0.285
**λ step**	-	-	0.1	0.1
**Maximum λ**	-	-	1.4	0.1

*HAR incorporating a cluster of point charges and dipoles.

Computational and experimental details

The AIMALL software[2] was used for performing QTAIM analysis and extract topological properties, atomic charges, and atomic dipole moments. Visualization of Intermolecular Interactions and MOGUL analysis was done using the Mercury software[3]. The VESTA software[4] aided us to visualize the interaction density and interaction ESP. Hirshfeld surfaces and fingerprints were obtained using CrystalExplorer 21.[5]

Intermolecular interactions and Hirshfeld Surface Analysis

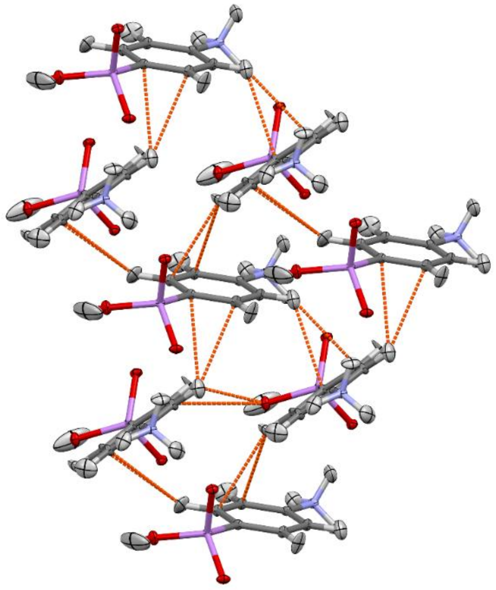

(a)

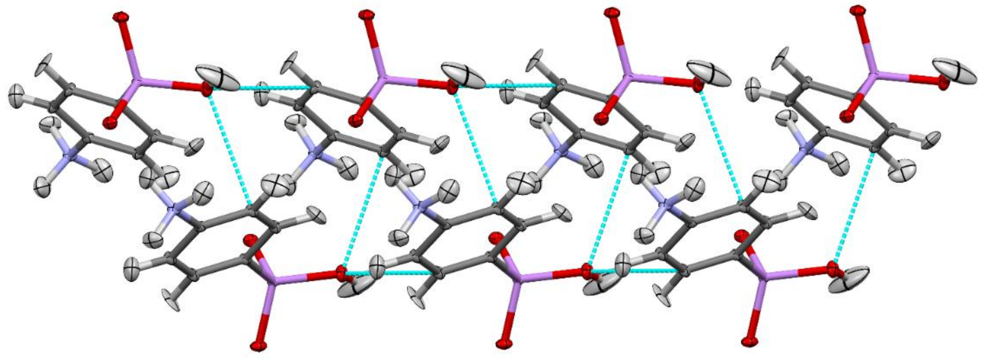

(b)

Figure S3. (a) CH-π interactions visualized with dashed orange lines and **(b)** lone pair-π interactions visualized with dashed blue lines inside the crystal packing of *p*-arsanilic acid. White, gray, blue, red, and purple atoms represent hydrogen, carbon, nitrogen, oxygen, and arsenic atoms, respectively.

Table S6. Distances of intermolecular interactions from atoms in the central molecule to symmetry-generated molecules in dataset 5. The shortest hydrogen bonds are given in more detail in the below table again.

**Hydrogen bonds**			**CH-π interactions**			**Lone pair - π interactions**		
**From**	**To**	**Å**	**From**	**To**	**Å**	**From**	**To**	**Å**
As1O1	H1b	1.690	H2	C5	2.996	C3	O3	3.301
	H1c	1.641		C3	2.901	C6	O3	3.241
AS1O2	H1a	1.685		C4	2.915	O3	C3	3.301
	H3	1.650	C3	H2	2.901	O3	C6	3.241
	H6	2.711	C4	H2	2.915			
As1O3	H3a	2.493	C5	H5	2.978			
O3H3	O2	1.650	H5	C5	2.978			
C3H3a	O3	2.493	C5	H2	2.996			
C6H6	O2	2.711						
N1H1a	O2	1.685						
N1H1b	O1	1.690						
N1H1c	O1	1.641						

**Donor**	**Hydrogen**	**Acceptor**	**D-H (Å)**	**H…A (Å)**	**D…A (Å)**	**D-H…A (˚)**	**Symmetry**
N1	H1a	O2	1.031(12)	1.685(13)	2.712(1)	173.0(1)	1-X, -1/2+Y, 1-Z
N1	H1b	O1	1.026(11)	1.690(11)	2.716(1)	178.7(1)	2-X, -1/2+Y, 1-Z
N1	H1c	O1	1.038(14)	1.641(13)	2.670(1)	171.2(1)	X, Y, 1+Z
O3	H3	O2	0.962(18)	1.650(17)	2.598(1)	169.1(2)	1-X, 1/2+Y, -Z

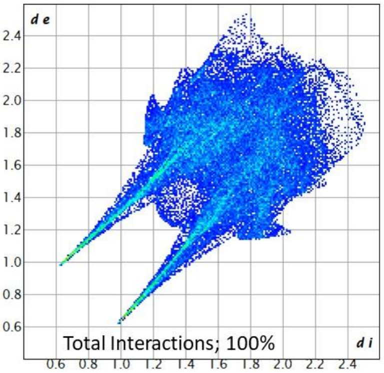

(Total)

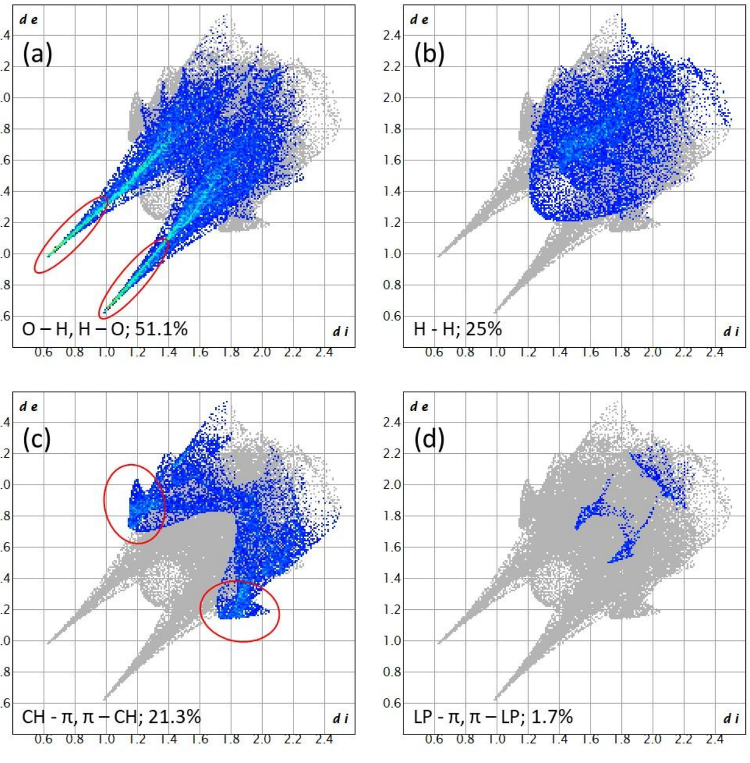

Figure S4. Fingerprint plot of the Hirshfeld surface of *p*-arsanilic acid showing the total interactions; (a) hydrogen bonds (O-H contacts) with their typical spikes encircled in red, 51.1%; (b) H...H interactions, 25%; (c) CH–π interactions (H-C contacts) with the typical chicken wings encircled in red, 21.3%; (d) lone pair (LP)–π interactions (O-C contacts), 1.7%.

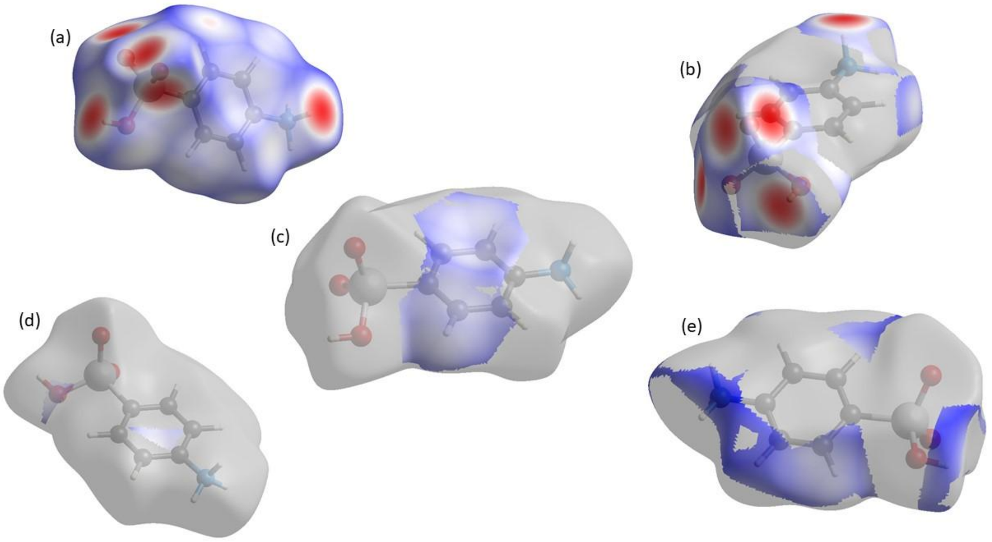

Figure S5. Hirshfeld surface of p-arsanilic acid with the property d_{norm} mapped onto it. Red color represents contacts for which the contact distance mediated by the surface is smaller than the sum of the van-der-Waals radii of the involved atoms. (a) total Hirshfeld surface color-coded, (b) hydrogen bonding matching with Figure S4a, (c) CH–π interactions matching with Figure S4c, (d) LP–π interaction matching with Figure S4d, (e) H...H interactions matching with Figure S4b.

Evaluation of p-arsanilic acid's geometry in a lysozyme crystal structure

In this section, we try to find out whether the atomically resolved structures of the three independent p-arsanilic acid molecules inside the protein are reliable. The three molecules ASR 140-142 were extracted from the protein crystal structure with PDB code 1N4F. The resolution of the crystal structure is 1.78 Å, close to atomic resolution. We further checked the PDB validation report to evaluate the p-arsanilic acid geometries inside the lysozyme structure (Table S7). Both AltConf (Alternate Conformation) and ZeroOcc (Zero Occupancy) parameters for all atoms of p-arsanilic acid are zero. AltConf=0 means that the ligand is not disordered or does not have multiple conformations. ZeroOcc=0 means that there is no atom in the ligand group with zero occupancy. These two parameters imply that the ligands' fit to the electron density map is reliable. The RSCC (Real Space Correlation Coefficient) measures how well the atomic model fits the experimental electron density map. All three molecules have parameters around or above 90%, which again indicates a good fit. The RSR (Real Space R-factor) parameter represents the discrepancy between observed and calculated electron density. RSR values smaller than 0.2 indicate a good model fit. For all three *p*-arsanilic acid molecules, the values are below 0.2. The B-factors (overall atomic displacement parameters, Å^2) indicate the atomic mobility or disorder in the structure. Low B-factors imply stable, well-

ordered atoms while high B-factor indicate flexible regions, poor electron density, or potential modeling errors. For p-arsanilic acid, the range of B factors is smaller than 50 $Å^2$ which shows that all three molecules are in a well-resolved regions not in flexible or disordered regions. Finally, Q < 0.9 (Quality Metric) lists the number of atoms with occupancy less than 0.9 and can suggest partial binding or uncertainty in position. In the case of ASR 140 (p-arsanilic acid residue number 140), Q < 0.9 is zero meaning that there is no atom in that ligand with occupancy less than 90%. However, the other two molecules do consist of only partially occupied atoms.

Table S7. Validation parameters for *p*-arsanilic acid ligands in the lysozyme crystal structure

Ligand	Number of Atoms	AltConf	ZeroOcc	RSCC	RSR	B-factors ($Å^2$)	Q<0.9
ASR 141	11	0	0	0.88	0.17	33,36,37,37	11
ASR 142	11	0	0	0.90	0.13	38,38,39,40	11
ASR 140	11	0	0	0.95	0.10	26,28,34,35	0

We further checked the geometrical parameters of each *p*-arsanilic acid ligand by means of a Mogul analysis. The Mogul geometry check is a tool to validate a particular molecule's three-dimensional conformation. This tool employs data from CCDC to check what are the range and magnitude of the values of a particular bond, angle, torsion, or ring a molecule can adopt. The result of this analysis aids us in identifying inconsistencies within a structure. It should be noted that this analysis is part of the PDB validation report, but we needed more detail from it, therefore we performed the analysis again. Table S8 collects the data from the Mogul analysis. In the table we have the magnitude (query value) of bond lengths, angles, and torsion angles which are unusual, and kind of outlier based on Mogul analysis. It should be noted that the other bond lengths, angles, and torsion that are not listed in the table are in the normal range and are not outliers. It is worthwhile to mention that, unlike small-molecule structures, the atomic coordinated in a PDB file do not have estimated standard uncertainties.[6,7] The standard uncertainties in the table are from the Mogul analysis, not from the protein crystal structure refinement.

If the value of the |z-score| is 3 or more (Table S8), that geometrical parameter is suspicious, meaning that it is not common compared to the other similar structures. Therefore, Mogul analysis considers that parameter as an outlier. However, it does not mean necessarily that this parameter is incorrect; it might be an unknown geometrical parameter for that type of structure. The other parameter, |d(min)|, is the difference between the value of the bond length, angle, or torsion angle in our query and the nearest value to our query in the Mogul distribution. All three *p*-arsanilic acids have outlier values and specifically we cannot prioritize them over each other. As we have shown before, ARS 140 has the best fit to the

electron density, discussed above, and here ARS 142 has the minimum number of outliers. However, the geometrical parameters of all three ligands are quite similar. Due to the better fit to the experimental electron density, we have chosen ARS 140 to evaluate the geometry of *p*-arsanilic acid while interacting with a protein in a biological environment for the analyses presented in the main paper.

Table S8. Mogul analysis parameters for bond lengths (Å), bond angles (°), and torsion angles (°) of all three *p*-arsanilic acid ligands in the lysozyme crystal structure.

Ligand	Fragment	Query value	Mean	Std. dev.	|z-score|	|d(min)|
ARS 140	As-C_1	1.950	1.906	0.018	2.453	0.012
	C_5-C_4	1.454	1.396	0.021	2.79	0
	C_4-N_7	1.501	1.376	0.036	3.454	0
	C_6-C_5	1.427	1.384	0.018	2.476	0
	O_2-AS-C_1	112.58	107.327	2.543	2.066	1.292
	O_2-AS-C_1-C_6	-120.721	-	-	-	10.286
ARS 141	As-C_1	1.951	1.906	0.018	2.503	0.013
	C_5-C_4	1.440	1.396	0.021	2.13	0
	C_4-N_7	1.493	1.376	0.036	3.217	0
	C_6-C_5	1.432	1.384	0.018	2.74	0
	O_1-AS-C_1-C_2	-55.304	-	-	-	10.906
ARS 142	C_5-C_4	1.438	1.396	0.021	2.054	0
	C_4-N_7	1.492	1.376	0.036	3.191	0
	C_6-C_5	1.429	1.384	0.018	2.586	0
	O_2-AS-C_1-C_6	-122.294	-	-	-	11.86

We further analyse how the three *p*-arsanilic acid molecules (ARS 140, ARS 141, ARS 142) bind with the protein. The interactions are predicted using PLIP 2021[8] (Figure S6). Since the hydrogen atoms are missing in the protein crystal structure, we do not have information regarding the hydrogen bonding. However, we can assume from the non-hydrogen atom positions that, in all three cases, *p*-arsanilic acid is bonded to protein residues through hydrogen bonds or water bridges involving the arsenite and ammonium groups. In the case of ARS 141, there is also a π-π interaction between the benzene ring of p-arsanilic acid and the ring of a proline amino acid. Table S9 summarizes the geometrical data related to these interactions.

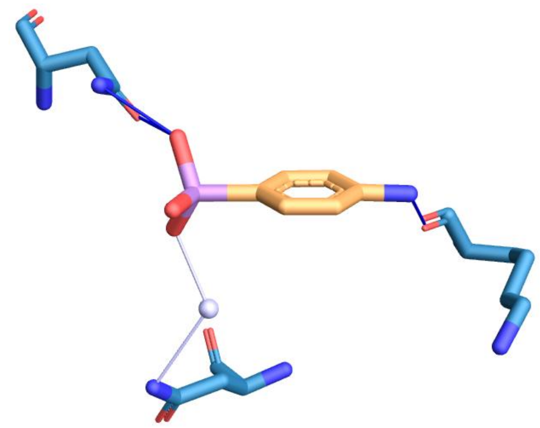

ARS 140

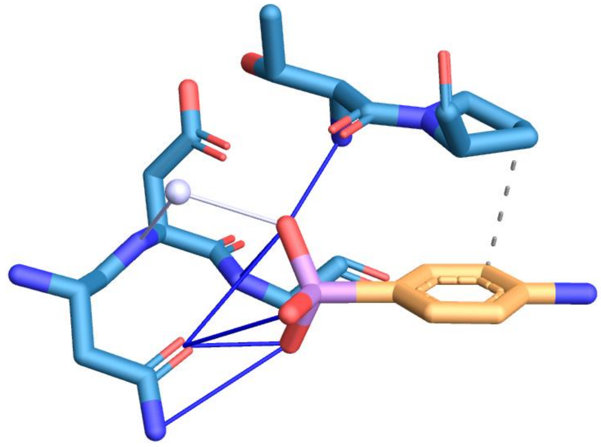

ARS 141

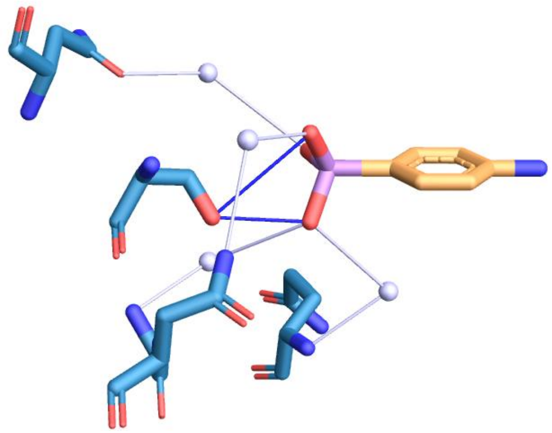

Ars 142

Figure S6. Representation of p-arsanilic acid's interactions with different protein residues in the crystal structure of lysozyme. The red and blue atoms represent oxygen and nitrogen, respectively. The white atoms represent water molecules. The pictures are generated using the PLIP 2021 web server.

Table S9. Geometrical parameters of the intermolecular interactions between *p*-arsanilic acid ligands and lysozyme. Distances in Å, angles in °. These data are generated using the PLIP 2021 web server that estimates hydrogen atom positions.

Part (A)

Interaction type	p-arsanilic acidnumber	residue	Amino acid	DIST H-A	DIST D-A	DON ANGLE	Donor atom	Acceptor atom
Hydrogen bond	ARS 140	33A	LYS	2.43	3.14	128.39	1024 [Npl]	252 [O2]
		44A	ASN	2.21	2.98	134.86	346 [Nam]	1015 [O3]
		44A	ASN	2.79	3.11	100.58	1015 [O3]	345 [O2]
	ARS 141	65A	ASN	3.33	3.79	110.56	525 [Nam]	1027 [O3]
		65A	ASN	1.55	2.52	173.96	1027 [O3]	524 [O2]
		65A	ASN	3.35	3.88	116.96	1028 [O3]	524 [O2]
		65A	ASN	3.27	3.83	118.65	1026 [O3]	524 [O2]
		67A	GLY	3.06	3.49	107.66	534 [Nam]	1026 [O3]
		69A	THR	1.76	2.69	155.70	549 [Nam	1026 [O3]
	ARS 142	24A	SER	2.10	2.98	149.17	191 [O3]	1037 [O3]
		24A	SER	2.03	2.98	163.34	1037 [O3]	191 [O3]
		24A	SER	3.15	3.82	127.99	1038 [O3]	191 [O3]

DIST H-A: Distance between hydrogen atom and acceptor; **DIST D-A:** distance between donor and acceptor of the hydrogen bond; **DON ANGLE:** angle between D-H…A.

Part (B)

Interaction type	p-arsanilic acid number	residue	Amino acid	DIST A-W	DIST D-W	Donor angle	WATER ANGLE	Donor atom	Acceptor atom	Water Atom
Water bridge	ARS 140	39A	ASN	2.66	3.21	150.31	123.75	310 [Nam]	1016 [O3]	1080
	ARS 141	66A	ASP	2.66	2.85	169.39	100.77	526 [Nam]	1026 [O3]	1097
	ARS 142	19A	ASN	3.01	3.88	131.91	123.08	1039 [O3]	145 [O2]	1179
		26A	GLY	3.80	2.99	144.02	124.90	200 [Nam]	1037 [O3]	1069
		27A	ASN	2.78	3.42	107.93	97.40	211 [Nam]	1038 [O3]	1076
		121A	GLN	3.67	3.08	161.61	85.28	935 [Nam]	1037 [O3]	1152

DIST A-W: Distance of the acceptor atom to the bridging water; **DIST D-W:** Distance of the donor atom to the bridging water

Part (C)

Interaction type	p-arsanilic acidnumber	residue	Amino acid	distance	Ligand atom	Protein atom
π-π (hydrophobic interactions)	ARS 141	70A	PRO	3.68	1031	561

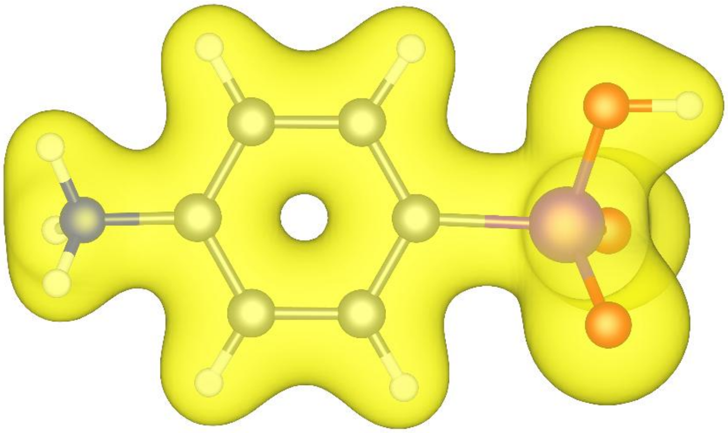

Figure S7. An isosurface representation at the value of 0.09 e/Å^3 of the electron density grid for the average of HF-XCW and DFT-XCW grids. We expect that this averaging eliminates the underestimation of the electron correlation effect in HF and the overestimation of electron correlation in DFT. This idea needs to be investigated further, but it is beyond the scope of this paper.

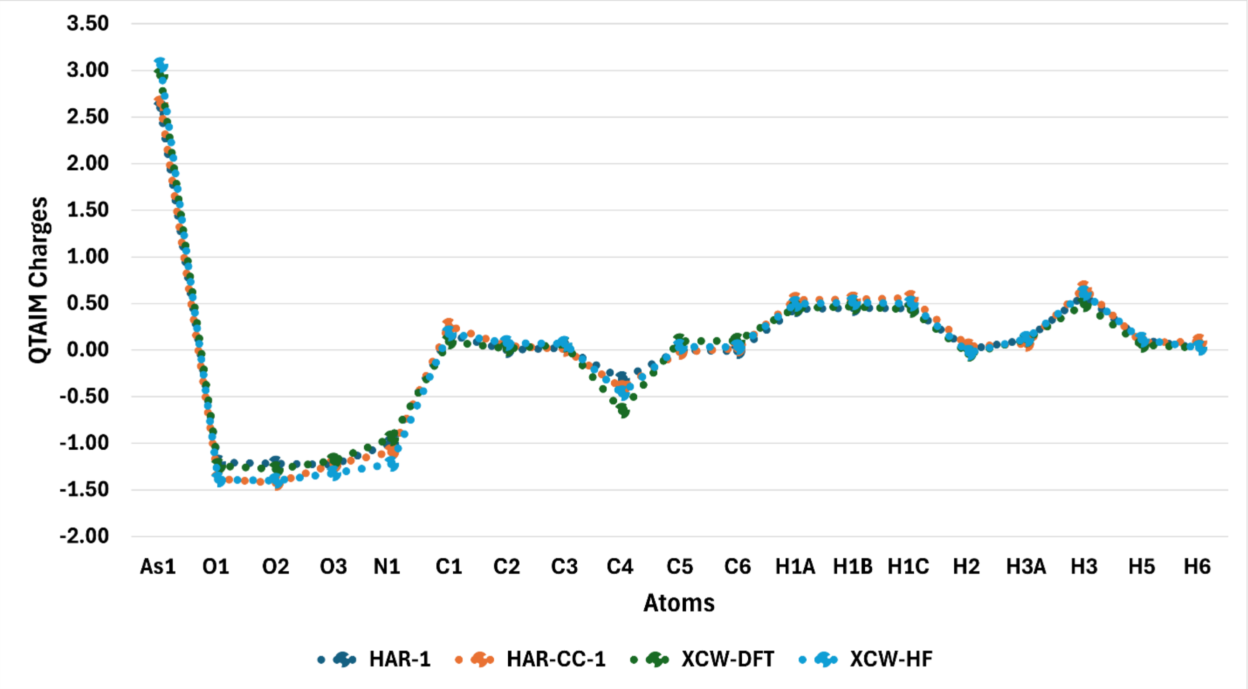

Figure S8. QTAIM charges (in e) of each atom (atomic basin) for our models in the first strategy with fixed geometry.

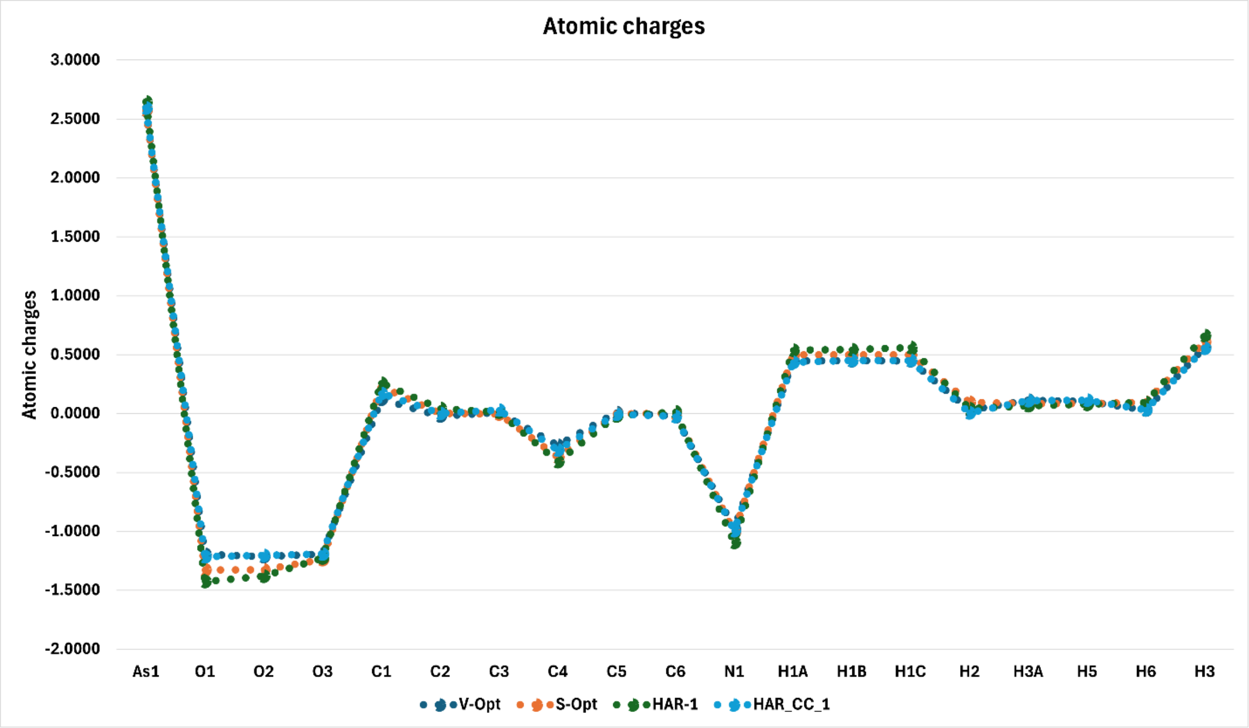

Atomic charges
3.0000
2.5000
2.0000
1.5000
1.0000
0.5000
0.0000
-0.5000
-1.0000
-1.5000
-2.0000
Atomic charges
As1
O1
O2
O3
C1
C2
C3
C4
C5
C6
N1
H1A
H1B
H1C
H2
H3A
H5
H6
H3
V-Opt
S-Opt
HAR-1
HAR_CC_1

(a)

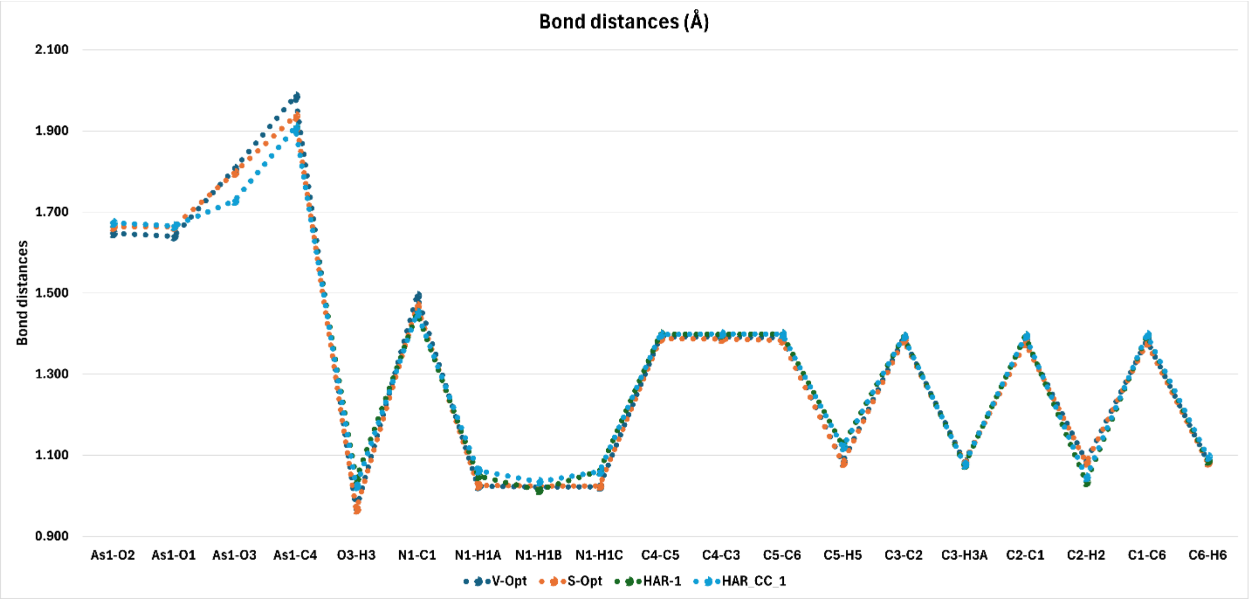

Bond distances (Å)
2.100
1.900
1.700
1.500
1.300
1.100
0.900
Bond distances
As1-O2
As1-O1
As1-O3
As1-C4
O3-H3
N1-C1
N1-H1A
N1-H1B
N1-H1C
C4-C5
C4-C3
C5-C6
C5-H5
C3-C2
C3-H3A
C2-C1
C2-H2
C1-C6
C6-H6
V-Opt
S-Opt
HAR-1
HAR_CC_1

(b)

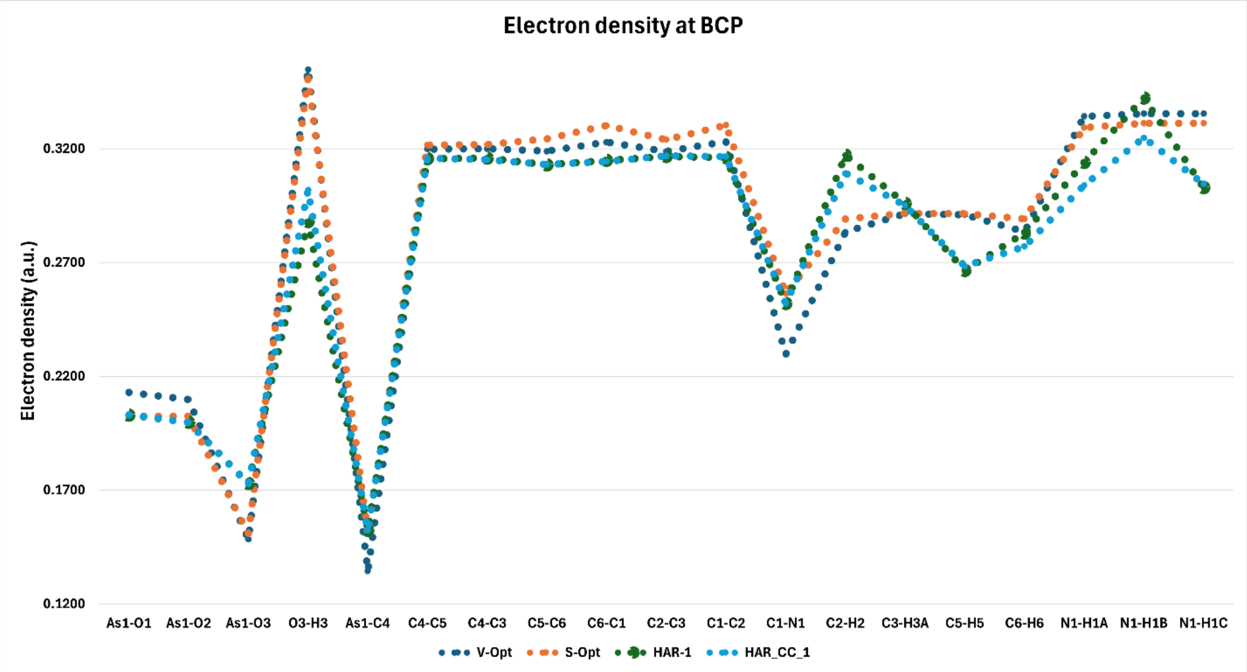

(c)

Figure S9. (a) Total atomic QTAIM charges (in e), **(b)** bond distances (in Å), and **(c)** electron density values at bond critical points (in a.u.), related to the models of the second strategy (flexible geometry).

Table S10. (a) Electron density at bond critical points (BCPs), **(b)** total atomic dipole moment, **(c)** total atomic QTAIM charges, and **(d)** bond distances related to the models of the second strategy (flexible geometry).

(a)

Electron Density at BCP (e/bohr^3)				
**Model**	**V-Opt**	**S-Opt**	**HAR-1**	**HAR_CC_1**
**As1-O1**	0.213	0.202	0.203	0.203
**As1-O2**	0.209	0.202	0.199	0.199
**As1-O3**	0.148	0.150	0.173	0.172
**O3-H3**	0.354	0.351	0.288	0.301
**As1-C4**	0.134	0.150	0.152	0.152
**C4-C5**	0.319	0.321	0.315	0.315
**C4-C3**	0.320	0.321	0.315	0.315
**C5-C6**	0.318	0.324	0.312	0.313
**C6-C1**	0.322	0.330	0.314	0.314
**C2-C3**	0.318	0.324	0.316	0.316
**C1-C2**	0.323	0.330	0.316	0.316
**C1-N1**	0.229	0.256	0.252	0.252
**C2-H2**	0.283	0.289	0.317	0.309
**C3-H3A**	0.291	0.291	0.296	0.295

**C5-H5**	0.291	0.291	0.266	0.268
**C6-H6**	0.283	0.289	0.283	0.277
**N1-H1A**	0.334	0.329	0.314	0.303
**N1-H1B**	0.335	0.331	0.342	0.324
**N1-H1C**	0.335	0.331	0.302	0.304

(b)

Total atomic dipole moment (Debye)				
**Model**	**V-Opt**	**S-Opt**	**HAR-1**	**HAR_CC_1**
**As1**	4.501	6.556	5.369	5.369
**O1**	4.211	5.139	4.418	4.414
**O2**	4.340	5.134	4.381	4.378
**O3**	2.610	2.645	2.595	2.575
**C1**	2.896	3.713	3.039	3.032
**C2**	1.809	2.544	1.867	1.868
**C3**	1.951	2.833	2.018	2.021
**C4**	5.352	6.719	5.243	5.237
**C5**	1.947	2.833	1.948	1.949
**C6**	1.829	2.543	1.874	1.878
**N1**	3.365	4.004	3.298	3.311
**H1A**	0.173	0.250	0.193	0.203
**H1B**	0.164	0.244	0.168	0.187
**H1C**	0.165	0.243	0.220	0.215
**H2**	0.335	0.172	0.398	0.386
**H3A**	0.165	0.199	0.161	0.158
**H5**	0.185	0.200	0.140	0.140
**H6**	0.333	0.171	0.339	0.329
**H3**	0.087	0.160	0.211	0.190

(c)

Total atomic QTAIM charge (e)				
**Model**	**V-Opt**	**S-Opt**	**HAR-1**	**HAR_CC_1**
**As1**	2.574	2.578	2.646	2.594
**O1**	-1.194	-1.327	-1.426	-1.215
**O2**	-1.214	-1.325	-1.381	-1.204
**O3**	-1.193	-1.238	-1.226	-1.191
**C1**	0.120	0.228	0.258	0.170
**C2**	-0.021	0.001	0.042	0.001
**C3**	0.009	-0.002	0.011	0.029
**C4**	-0.269	-0.368	-0.408	-0.308

**C5**	0.009	-0.002	-0.019	-0.005
**C6**	-0.022	0.001	0.014	-0.024
**N1**	-0.978	-1.009	-1.093	-0.994
**H1A**	0.445	0.498	0.537	0.434
**H1B**	0.448	0.499	0.542	0.451
**H1C**	0.447	0.499	0.560	0.450
**H2**	0.029	0.095	0.040	0.006
**H3A**	0.113	0.084	0.066	0.110
**H5**	0.101	0.084	0.078	0.113
**H6**	0.029	0.095	0.093	0.0315
**H3**	0.564	0.609	0.662	0.554

(d)

**Bond distance (Å)**				
**Model**	**V-Opt**	**S-Opt**	**HAR-1**	**HAR_CC_1**
**As1-O2**	1.648	1.664	1.673	1.673
**As1-O1**	1.640	1.663	1.665	1.665
**As1-O3**	1.807	1.797	1.727	1.727
**As1-C4**	1.985	1.937	1.909	1.909
**O3-H3**	0.966	0.966	1.042	1.024
**N1-C1**	1.493	1.469	1.452	1.452
**N1-H1A**	1.024	1.026	1.049	1.063
**N1-H1B**	1.022	1.024	1.014	1.035
**N1-H1C**	1.022	1.024	1.062	1.060
**C4-C5**	1.392	1.387	1.399	1.398
**C4-C3**	1.392	1.387	1.399	1.399
**C5-C6**	1.390	1.383	1.399	1.399
**C5-H5**	1.083	1.081	1.124	1.120
**C3-C2**	1.390	1.384	1.393	1.393
**C3-H3A**	1.083	1.081	1.076	1.077
**C2-C1**	1.383	1.377	1.395	1.395
**C2-H2**	1.085	1.082	1.034	1.045
**C1-C6**	1.384	1.377	1.396	1.397
**C6-H6**	1.085	1.082	1.086	1.096

References

(1) Y. Balmohammadi, L. A. Malaspina, Y. Nakamura, G. Cametti, M. Siczek and S. Grabowsky, *Sci. Rep.*, 2025, **15**, 13584.

(2) T. A. Keith, AIMAll (Version 13.05. 06). *TK Gristmill Software: Overland Park, KS, USA* **2013**.

(3) C. F. Macrae, I. Sovago, S. J. Cottrell, P. T. A. Galek, P. McCabe, E. Pidcock, M. Platings, G. P. Shields, J. S. Stevens, M. Towler, P. A. Wood, *J. Appl. Crystallogr.* 2020, **53** (1), 226–235.

(4) K. Momma, F. Izumi, *J. Appl. Crystallogr.* 2008, ***41*** (3), 653–658.

(5) P. R. Spackman, M. J. Turner, J. J. McKinnon, S. K. Wolff, D. J. Grimwood, D. Jayatilaka, M. A. Spackman, *J. Appl. Crystallogr.* 2021, ***54*** (3), 1006–1011.

(6) M. Gurusaran, M. Shankar, R. Nagarajan, J. R. Helliwell, K. Sekar. *IUCrJ* 2014, ***1*** (1), 74–81.

(7) J. R. Helliwell, *Curr. Res. Struct. Biol.*, 2023, ***6***, 100111.

(8) M. F. Adasme, K. L. Linnemann, S. N. Bolz, F. Kaiser, S. Salentin, V. J. Haupt, M. Schroeder, *Nucleic Acids Res.* 2021, ***49*** (W1), W530–W534.
